# Endothelial deletion of *Ino80* disrupts coronary angiogenesis and causes congenital heart disease

**DOI:** 10.1038/s41467-017-02796-3

**Published:** 2018-01-25

**Authors:** Siyeon Rhee, Jae I. Chung, Devin A. King, Gaetano D’amato, David T. Paik, Anna Duan, Andrew Chang, Danielle Nagelberg, Bikram Sharma, Youngtae Jeong, Maximilian Diehn, Joseph C. Wu, Ashby J. Morrison, Kristy Red-Horse

**Affiliations:** 1 Department of Biology, Stanford University, 371 Serra Mall, Stanford, CA 94305 USA; 2 Stanford Cardiovascular Institute, Stanford University School of Medicine, Stanford, CA 94305 USA; 3 Department of Medicine, Stanford University School of Medicine, Stanford, CA 94305 USA; 4 Institute for Stem Cell Biology and Regenerative Medicine, Stanford University School of Medicine, Stanford, CA 94305 USA; 5 Stanford Cancer Institute, Stanford University School of Medicine, Stanford, CA 94305 USA; 6 Department of Radiation Oncology, Stanford University School of Medicine, Stanford, CA 94305 USA

## Abstract

During development, the formation of a mature, well-functioning heart requires transformation of the ventricular wall from a loose trabecular network into a dense compact myocardium at mid-gestation. Failure to compact is associated in humans with congenital diseases such as left ventricular non-compaction (LVNC). The mechanisms regulating myocardial compaction are however still poorly understood. Here, we show that deletion of the Ino80 chromatin remodeler in vascular endothelial cells prevents ventricular compaction in the developing mouse heart. This correlates with defective coronary vascularization, and specific deletion of Ino80 in the two major coronary progenitor tissues—sinus venosus and endocardium—causes intermediate phenotypes. In vitro, endothelial cells promote myocardial expansion independently of blood flow in an Ino80-dependent manner. Ino80 deletion increases the expression of E2F-activated genes and endothelial cell S-phase occupancy. Thus, *Ino80* is essential for coronary angiogenesis and allows coronary vessels to support proper compaction of the heart wall.

## Abstract (web summary)

Heart development requires compaction of the ventricular wall into a dense myocardium at mid-gestation. Here, Rhee and colleagues show that the chromatin remodeller Ino80 is critical for the formation of the coronary vasculature, and show that coronary vessels are needed for successful cardiac compaction during embryonic development.

## Introduction

Morphogenic events that give tissues their appropriate shape during embryonic development are an important aspect of organ maturation, and defects in this process often underlie congenital malformations. One critical morphogenic process during heart development is myocardial compaction, which occurs when the ventricular wall is changed from being mostly trabecular (i.e., consisting of finger-like projections) to a thick, densely compacted muscle layer^1–3^. This involves proliferation and expansion of cardiomyocytes in the compact myocardium in the outer heart wall, and the coalescence of trabeculae in the innermost heart wall^4–6^. Compaction is important for the heart to function properly, which is underscored by the observation that defects in this process result in human cardiomyopathy. For example, left ventricular non-compaction (LVNC) is the third most common cardiomyopathy and results when the compact myocardium remains abnormally thin with expanded trabeculae, which can compromise heart function^1, 7^. How LVNC arises is not well understood; however, it is thought to develop during embryogenesis^8, 9^. Thus, understanding myocardial compaction during embryonic development could have implications for human disease.

Multiple mouse models have demonstrated that defective coronary vessel development is accompanied by abnormal growth of the compact myocardium^10–14^; however, a detailed analysis on the role of coronary vessels during myocardial compaction has not been performed. Coronary vessels would be required to bring blood flow to growing cardiac tissue. However, there is also mounting evidence that blood vessels secrete proteins, termed angiocrines, that affect the growth, survival, and differentiation of adjacent cells, independent of oxygenation^15, 16^. Interestingly, the mouse heart possesses at least two endothelial progenitor pools for their coronary vascular bed, the sinus venosus and endocardium^4, 14, 17, 18^. How the existence of two progenitor populations would influence the myocardial compaction process, and whether this involves blood vessel-derived signals, in addition to oxygenation, is not known.

It has been reported that human mutations in the Ino80 chromatin remodeler complex correlate with cardiovascular disease^19^, and we sought to investigate its role during cardiac development. Ino80 is an evolutionarily conserved, multisubunit chromatin remodeler that regulates transcription by positioning nucleosomes at target genes^20, 21^. The complex is named for the Ino80 ATPase subunit that catalyzes nucleosome rearrangements. The activity and structure of the Ino80 complex has been well-studied in highly purified experimental systems^22^. In *S. cerevisiae*, the complex plays diverse roles in DNA-templated processes^23–27^, including metabolic gene expression regulation^28^. In mammalian cells, the Ino80 complex has been shown to inhibit embryonic stem cell (ESC) differentiation through the maintenance of ‘open’ chromatin at pluripotent gene promoters^29, 30^, and contribute to tumorigenesis by increasing the accessibility of enhancers in cancer cells^31^. Although these data clearly show important roles for Ino80-mediated chromatin regulation, it is not known whether, in multicellular organisms, it is required in all cells or in specific contexts. *Ino80*-deficient mice have been generated, but do not undergo gastrulation, prohibiting analysis of its role during organogenesis^32^. Therefore, tissue specific deletions of *Ino80* are needed to assess its role during tissue and organ formation.

Here, we discovered that deleting the *Ino80* chromatin remodeler from embryonic endothelial cells results in ventricular non-compaction. Coronary vascularization was dramatically decreased in *Ino80* mutants while Ino80 inhibited E2F target gene expression and endothelial cells S-phase occupancy. In vitro assays showed that coronary endothelial cells support myocardial growth in a blood flow-independent manner, ultimately supporting a model where endothelial Ino80 is required for coronary vessels to expand and support myocardial compaction.

## Results

### *Ino80* endothelial deletion causes ventricular non-compaction

**Fig. 1:**
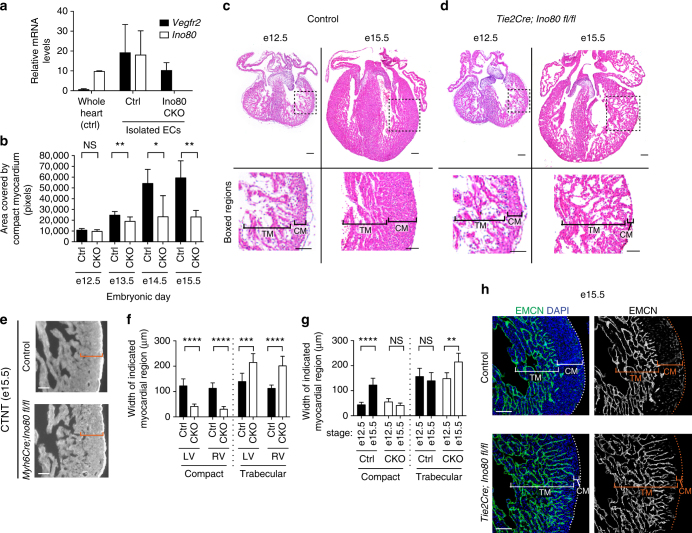
Compact myocardium development is disrupted with endothelial-specific knockout of *Ino80*. **a** Quantitative PCR analysis of endothelial cells (ECs) isolated from control (Ctrl) and *Tie2Cre;Ino80 fl/fl* (CKO) hearts reveal that *Vegfr2* is expressed while *Ino80* mRNA is undetectable. Error bars in graphs are standard deviation. (control, *n* = 3 hearts; mutant, *n* = 3 hearts). **b** Measurements of the area covered by compact myocardium in tissue sections from control or *Tie2Cre;Ino80 fl/fl* hearts at the indicated embryonic stages. NS nonsignificant, *P < 0.05; **P < 0.01, evaluated by Student’s *t*-test. **c**, **d** Tissue sections stained with Hematoxylin and Eosin show trabecular myocardium (TM) and compact myocardium (CM) in control (**c**) and CKO hearts (**d**). Images are representative of the following number of replicates: control, *n* = 6 hearts; mutant, *n* = 5 hearts at e12.5, control, *n* = 9 hearts; mutant, *n* = 6 hearts at e15.5. Scale bars: 100 μm. **e** Compact myocardium (orange brackets) is not thin with myocardial-specific deletion of *Ino80* (*Myh6Cre;Ino80 fl/fl)*. Images are representative of the following number of replicates: control, *n* = 6 hearts; mutant, *n* = 5 hearts. Scale bars: 100 μm. **f** Trabecular myocardium thickness is increased in the left and right ventricles in *Ino80* CKOs. Error bars in graphs are sd. (control, *n* = 6 hearts; mutant, *n* = 5 hearts at e12.5, control, *n* = 9 hearts; mutant, *n* = 6 hearts at e15.5). ***P < 0.001; ****P < 0.0001, evaluated by Student’s *t*-test. **g** The normal growth in compact layer thickness from e12.5 to 15.5 is replaced by an abnormal expansion of the trabecular layer in mutant hearts. Error bars in graphs are standard deviation. (control, *n* = 6 hearts; mutant, *n* = 5 hearts at e12.5, control, *n* = 9 hearts; mutant, *n* = 6 hearts at e15.5). NS nonsignificant, **P < 0.01; ****P < 0.0001, evaluated by Student’s *t*-test. **h** Endomucin (EMCN) immunofluorescence to label endocardium shows an expansion of this cell type into the area occupied by compact myocardium in control hearts. Images are representative of the following number of replicates: *n* = 9 hearts; mutant, *n* = 6 hearts at e15.5. Scale bars: 100 μm

To investigate the role of *Ino80-*mediated chromatin remodeling during cardiovascular development, we used a conditional knockout mouse line to delete *Ino80* in different cardiac cell types and analyzed the effects on heart development. The removal of Ino80 protein by Cre recombination in this mouse line was confirmed in isolated MEFs (Supplementary Fig. 1a, uncropped image in Supplementary Fig. 7). *Ino80* was expressed in multiple cell types in the heart (Supplementary Fig. 1b). We therefore used three Cre lines to individually delete the *Ino80* gene from either cardiomyocytes, the epicardium, or endothelial cells. The most apparent phenotype occurred in embryos with endothelial-specific deletions. In this cross, *Ino80* was deleted from all endothelial and endocardial cells using the *Tie2Cre* deleter line, which resulted in undetectable levels of *Ino80* mRNA in isolated endothelial cells (Fig. 1a). The resulting mutant mice displayed a dramatic cardiac phenotype that resembled ventricular non-compaction.

In wild-type mice during mid-gestation, the heart wall transitions from a mass of loosely associated trabeculae into a highly compact muscle layer in a process termed compaction. The compact layer grew dramatically as wild-type embryos progressed from e12.5 to e15.5 (Fig. 1b, c). However, in *Tie2Cre;Ino80 fl/fl* animals, the area occupied by compact myocardium plateaued at e13.5 (Fig. 1b, d). (Controls are all genotypes except *Tie2Cre;Ino80 fl/fl*, as heterozygous deletion did not exhibit a phenotype.) This phenotype was coincident with decreased survival between e15.5 and e16.5 (Supplementary Fig. 2). In contrast, deleting *Ino80* in cardiomyocytes using Myh6Cre did not affect compact myocardial growth during these stages (Fig. 1e, Supplementary Fig. 3), although we cannot rule out that this Cre may be activated too late to see a phenotype or in a mosaic fashion. Thus, these data indicate that Ino80 is particularly important in endothelial cells and that its presence is required to support compact myocardial growth.

Despite the very thin compact myocardium in the *Ino80* mutant mice, the heart wall contained large numbers of myocardial cells arranged into loosely packed trabeculae (Fig. 1d). Measuring the thickness of the trabecular and compact zones in heart sections showed that while the compact layer was greatly reduced, the trabeculae were significantly expanded in both the left and right ventricles (Fig. 1f). Furthermore, comparing these same measurements at two developmental time points, e12.5 and e15.5, revealed that the compact layer expanded in control animals as embryogenesis proceeded while the trabeculae width remained the same. However, in mutants, the opposite was observed: the compact layer growth was static while the trabeculae expanded (Fig. 1g). Endomucin immunofluorescence to highlight the endocardium, which is the inner lining of the heart, showed that these cells extended abnormally close to the surface of the heart in mutants (Fig. 1h). These data suggest that *Ino80* deletion leads to a thinned compact layer at the expense of increased trabeculation.

**Fig. 2:**
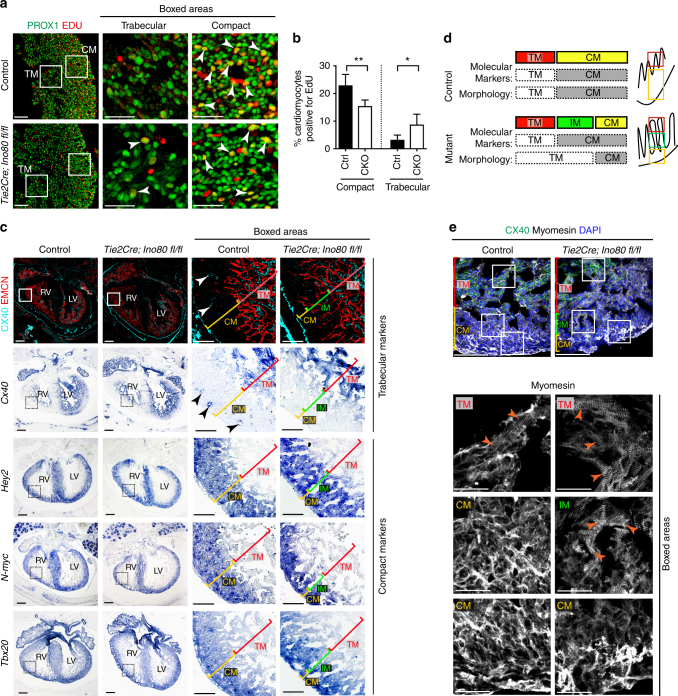
*Ino80* deletion in endocardial/endothelial cells results in ventricular non-compaction. **a** Tissue sections through e15.5 control and *Tie2Cre;Ino80 fl/fl* hearts treated with EdU and Immunostained for EdU and PROX1 to reveal proliferating cardiomyocytes (arrowheads). Images are representative of the following number of replicates: control, *n* = 7 hearts; mutant, *n* = 5 hearts at e15.5. Scale bars: 100 μm (low) and 50 μm (high magnification). **b** Quantification showed that compact myocardium proliferation is reduced while trabecular proliferation is increased. Error bars in graphs are standard deviation. (control, *n* = 7 hearts; mutant, *n* = 5 hearts at e15.5). *P < 0.05; **P < 0.01, evaluated by Student’s *t*-test. **c** Immunofluorescence and in situ hybridization on adjacent sections for trabecular (Cx40) and compact (Hey2, N-myc, and Tbox20) myocardium markers. Top row includes Endomucin (EMCN) immunofluorescence to demonstrate that mutants contain endocardial-lined trabeculae in regions where control hearts are compacted. Abnormal trabeculae adjacent to the thinned compact layer lack trabecular markers and express compact markers, a pattern that has been termed intermediate myocardium (IM). Images are representative of the following number of replicates: control, *n* = 3 hearts; mutant, *n* = 3 hearts at e15.5. Arrowheads indicate Cx40^+^ coronary arteries present in control hearts. Scale bars: 100 μm (low) and 25 μm (high magnification). **d** Schematic of marker expression and morphology in control and mutant hearts. Intermediate myocardium is a region exhibiting a mismatch in morphology and markers, and is only extensive in non-compacted hearts **e** Myomesin immunofluorescence reveals that intermediate myocardium also contains trabecular-like sarcomere morphology (arrowheads). Images are representative of the following number of replicates: control, *n* = 3 hearts; mutant, *n* = 3 hearts at e15.5. CM compact myocardium, TM trabecular myocardium. Scale bars: 100 μm (low) and 25 μm (high magnification)

To ascertain whether this phenotype could be due to ventricular non-compaction, we assessed cardiomyocyte proliferation and the expression patterns of compact and trabecular markers. Measuring EdU incorporation in cardiomyocytes using PROX1-specific antibodies^33^ revealed that mutants had decreased proliferation in the compact layer but increased proliferation in the trabecular layer at e15.5 (Fig. 2a, b). Immunofluorescence and/or in situ hybridization was next performed for compact layer markers (Hey2, Tbx20, and N-myc) and a trabecular marker (Cx40). This analysis revealed that the myocardium with trabecular morphology (loosely packed, endocardial-lined), which was adjacent to the thin compact layer of *Ino80* mutants, expressed the compact markers Hey2, Tbx20, and N-myc and lacked the trabecular marker, Cx40 (Fig. 2c). This was seen in both the left and right ventricles. The presence of myocardium with compact identity, but trabecular morphology is reminiscence of the “intermediate myocardium” previously described for non-compacted hearts lacking components of the NOTCH signaling pathway^10^. Intermediate myocardium is schematized in Fig. 2d and labeled in Fig. 2c, e. Immunofluorescence for the sarcomeric protein, Myomesin, revealed that highly structured sarcomeres were normally restricted to the CX40^+^ trabecular myocardium at e15.5. However, the trabecular-like Myomesin pattern was also present in the CX40-negative abnormal intermediate myocardium in non-compacted *Tie2Cre;Ino80 fl/fl* hearts (Fig. 2e). Taken together, these disruptions in heart wall patterning indicate that *Ino80* mutant mice exhibit ventricular non-compaction.

### Specific deletion in the endocardium and sinus venosus

**Fig. 3:**
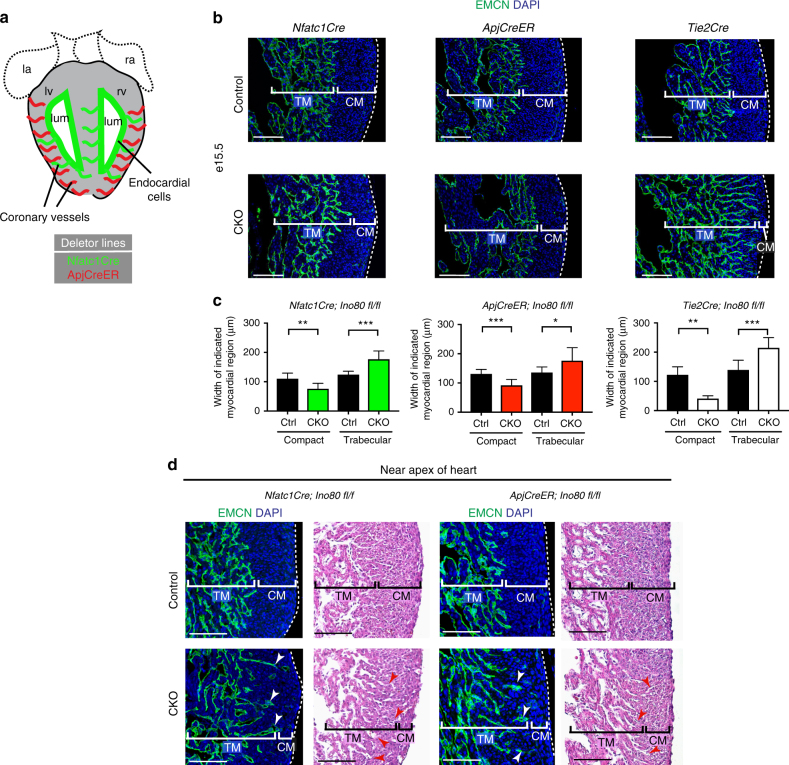
Deletion of *Ino80* using sinus venosus- or endocardial-specific Cre lines. **a** Schematic section through the heart showing the two types of endothelial cells within the heart and the Cre lines that differentially label each subset. **b** Endomucin (endocardial cells) and DAPI (nuclei) immunofluorescence in e15.5 hearts highlights reduction of compact myocardial thickness in hearts where *Ino80* was deleted using either *Nfatc1Cre*, *ApjCreER*, or *Tie2Cre*. Images are representative of the following number of replicates: *Nfatc1Cre* (control, *n* = 9 hearts; mutant, *n* = 6 hearts), *ApjCreER* (control, *n* = 12 hearts; mutant, *n* = 4 hearts), or *Tie2Cre* (control, *n* = 9 hearts; mutant, *n* = 6 hearts). Conditional knockouts are labeled CKO. Scale bars: 100 μm. **c** Quantification of compact and trabecular myocardial thickness in each condition. Error bars in graphs are standard deviation. *Nfatc1Cre* (control, *n* = 9 hearts; mutant, *n* = 6 hearts), *ApjCreER* (control, *n* = 12 hearts; mutant, *n* = 4 hearts), or *Tie2Cre* (control, *n* = 9 hearts; mutant, *n* = 6 hearts). *P < 0.05; **P < 0.01; ***P < 0.001, evaluated by Student’s *t*-test. **d** Example of abnormal extensions of Endomucin-positive endocardial cells (arrowheads) into the compact layer in CKO embryos. H&E sections are of comparable regions of the apex. Images are representative of the following number of replicates: *Nfatc1Cre* (control, *n* = 9 hearts; mutant, *n* = 6 hearts), *ApjCreER* (control, *n* = 12 hearts; mutant, *n* = 4 hearts). Scale bars: 100 μm

In the previously described experiments, *Ino80* was deleted from all endothelial cell types within the heart. We next investigated which subtype requires *Ino80* during cardiac development. There are two predominant types of endothelial cells in the heart: (1) endocardial cells that line the heart lumen and (2) coronary endothelial cells that vascularize ventricular muscle (Fig. 3a)^4, 14, 17, 18^. Endocardial cells are known to express Notch ligands and paracrine factors such as Nrg1 that support early myocardial trabeculation at e9.5 ^34, 1, 35^. Endocardial cells also support compact myocardial growth at later developmental stages through Notch signaling^10, 36^. Coronary vessels also influence myocardial growth, although the precise mechanisms involved are not well defined^10, 11^. We individually deleted *Ino80* in each of the two endothelial subsets: (1) endocardial cells using *Nfatc1Cre*^14^ (note that this line also deletes in endocardial-derived coronary vessels) and (2) sinus venosus (SV)-derived coronary vessels using *ApjCreER*^18^ (Fig. 3a). This experiment was designed to identify either the endocardial or coronary endothelial subset as requiring *Ino80* during myocardial compaction.

The results of this experiment were unexpected in that they showed that deleting *Ino80* in either subset affected compact myocardial growth. Measuring compact myocardial thickness in *Nfatc1Cre;Ino80 fl/fl* and *ApjCreER;Ino80 fl/fl* hearts revealed both Cre lines decreased myocardial thickness to a similar degree (average of 35–40 μm decrease in thickness), which was approximately half the magnitude of the full endothelial knockout using *Tie2Cre* deleter (average of 81 μm decrease in thickness; Fig. 3b, c). Furthermore, both *Nfatc1Cre;Ino80 fl/fl* and *ApjCreER;Ino80 fl/fl* resulted in expanded trabecular length (Fig. 3b, c), indicating an intermediate phenotype with each endothelial subtype. The non-compaction phenotype in the endothelial subset knockout lines was less severe and more heterogeneous than the pan-endothelial knockouts. Specifically, there was variability in the extent of the phenotype in different regions. For example, the apex of the heart was more susceptible to developing non-compact regions in both conditional knockout lines (Fig. 3d, compare to 3b). Interestingly, this regional pathology is more similar to the human disease than ventricle-wide non-compaction^7^. These data show that Ino80 functions collectively in both the SV-derived coronary vessels as well as the endocardial cells and/or endocardial-derived coronary vessels to support compact myocardial expansion.

### *Ino80* deletion results in defective angiogenesis

To ascertain the underlying cellular processes leading to ventricular non-compaction, we observed the structure of the endocardium and coronary vessels in different *Ino80* endothelial-specific conditional knockout lines. Hematoxylin and eosin histological staining and immunofluorescence for endothelial markers (VE-cadherin, VEGFR2) did not reveal obvious structural defects in the endocardium in any of the mouse lines (Fig. 1d). Also, early trabecular development (initiating at e9.5–10.5) is not affected in *Tie2Cre;Ino80 fl/fl* hearts indicating that early communication between the endocardial cells and myocardium is not compromised in the absence of *Ino80* (Fig. 1d). These analyses suggested that the structure and early functionality of the endocardium is normal.

**Fig. 4:**
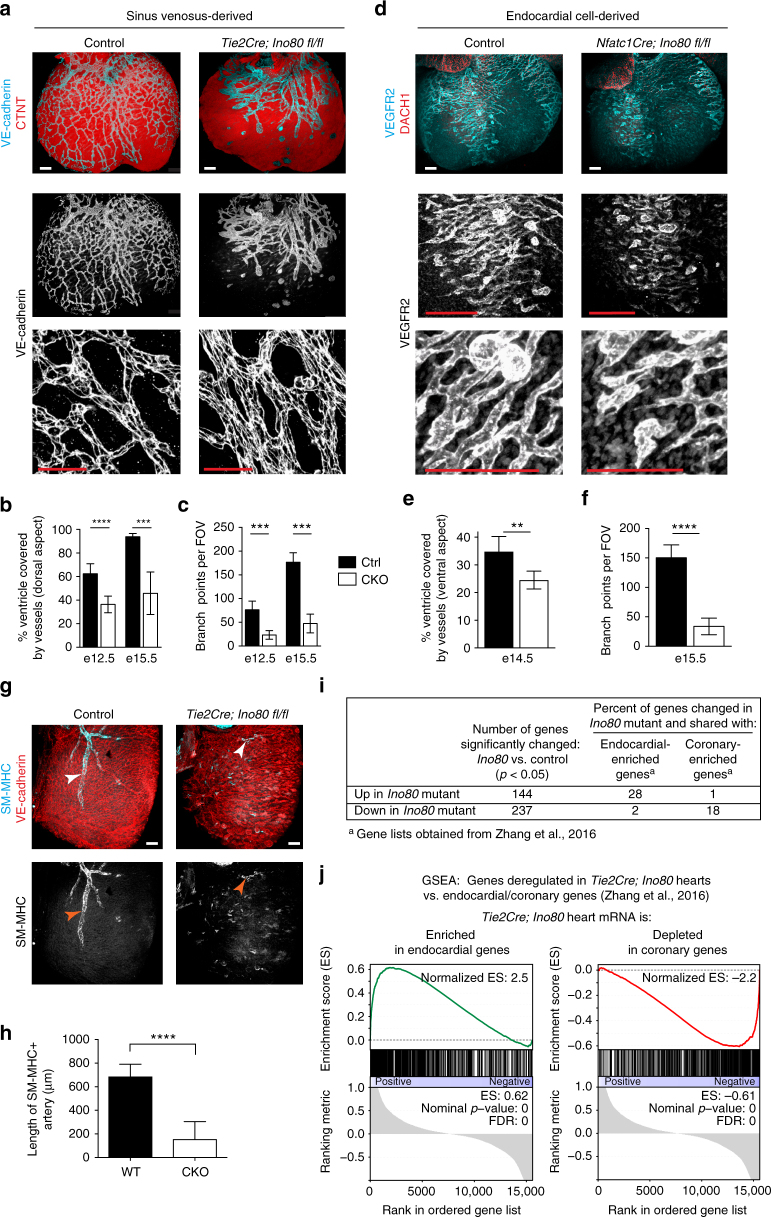
In vivo angiogenesis from both the sinus venosus and endocardial cells is defective with *Ino80* deletion. **a**–**f** Whole-mount confocal images (**a**, **d**) and quantification (**b**, **c**, **e**, **f**) of coronary vessel growth in indicated *Ino80*-deficient hearts. **a**, **b** VE-cadherin immunofluorescence (**a**) and quantification of heart coverage (**b**) revealed that vessel growth on the dorsal side of the heart where the sinus venosus sprouts is significantly stunted. Images are representative of the following number of replicates: control, *n* = 4 hearts; mutant, *n* = 7 hearts at e15.5. Error bars in graphs are sd. (control, *n* = 8 hearts; mutant *n* = 6 hearts at e12.5, control, *n* = 4 hearts; mutant, *n* = 7 hearts at e15.5). ****P < 0.0001, evaluated by Student’s *t*-test. Scale bars: 100 μm. **c** Vessel branching is also reduced in whole hearts. Error bars in graphs are sd. (control, *n* = 5 hearts; mutant, *n* = 6 hearts at e12.5, control, *n* = 4 hearts; mutant, *n* = 7 hearts at e15.5). ***P < 0.001, evaluated by Student’s *t*-test. **d**, **e** VEGFR2 immunofluorescence (**d**) and vessel coverage (**e**) shows a similar significant decrease on the ventral side of the heart where endocardial cell-derived coronary migration occurs. Images are representative of the following number of replicates: control, *n* = 6 hearts; mutant, *n* = 5 hearts at e15.5. Error bars in graphs are standard deviation. (control, *n* = 6 hearts; mutant, *n* = 5 hearts at e15.5). **P < 0.01, evaluated by Student’s *t*-test. Scale bars: 100 um. **f** Endocardial-derived vessel branching is also stunted in whole hearts. Error bars in graphs are standard deviation. (control, *n* = 4 hearts; mutant, *n* = 6 hearts at e15.5). ****P < 0.0001, evaluated by Student’s *t*-test. Scale bars: 100 μm. **g**, **h** Confocal images of the right lateral side of e15.5 hearts (**g**) and quantification (**h**) showed a dramatic decrease in SM-MHC^+^ arterial smooth muscle (arrowheads) in mutant hearts. Error bars in graphs are standard deviation. Images are representative of the following number of replicates: control, *n* = 5 hearts; mutant, *n* = 6 hearts. Scale bars: 100 μm. **i**,** j** Comparison of genes changed in e13.5 *Tie2Cre;Ino80 fl/fl* hearts with those reported to be specifically expressed in either endocardial or coronary endothelial cells. *Ino80* mutants are enriched in endocardial genes and depleted of coronary genes

In contrast to the endocardium, defects in coronary vessels were very apparent. To assess SV-derived angiogenesis, we observed coronary vessels in regions primarily derived from the SV. We used *Tie2Cre;Ino80 fl/fl* animals because they are expected to have more complete recombination^18^. Whole-mount immunostaining of embryonic hearts revealed that coronary vessel growth on the dorsal side of the heart was stunted in *Tie2Cre;Ino80 fl/fl* animals (Fig. 4a). The phenotype was observed even at e12.5 before the thin myocardium/non-compaction phenotype was apparent (Fig. 4b). The delayed vessels were also wider with fewer branch points than controls (Fig. 4a, c). We also observed defects in angiogenesis from the endocardial cells. Endocardial angiogenesis can be visualized on the ventral side of the heart where SV-derived vessels rarely contribute to the coronary vasculature^18, 37^. Normally, coronary vessels emerge from the midline and migrate laterally to populate the ventral side of the heart. We observed far fewer vessels in this location in *Tie2Cre;Ino80 fl/fl* and *Nfatc1Cre;Ino80 fl/fl* animals (Fig. 4d). Quantification of the extent of migration (Fig. 4e) and branching (Fig. 4f) of the ventral vessels revealed significant decreases in the mutants compared to controls. These defects in angiogenesis were also accompanied by dramatic decreases in the assembly of smooth muscle covered coronary arteries (Fig. 4g, h). These data demonstrate that vascularization from the two predominant coronary vessel progenitor sources is inhibited in the absence of *Ino80*.

The above data suggested that the ventricular non-compaction phenotype in *Ino80-*deficient mice could be secondary to defects in coronary sprouting, and we sought further evidence that angiogenesis was a predominating factor. mRNA sequencing was performed on whole e13.5 hearts from control and *Tie2Cre;Ino80 fl/fl* animals, a time point when the compact myocardium phenotype was apparent, but not yet severe, thus limiting non-cell autonomous effects of *Ino80* deletion (Fig. 1b). Comparing the expression changes between control and *Tie2Cre;Ino80 fl/fl* revealed that 144 genes were significantly upregulated and 237 were significantly downregulated in mutants. Cross-referencing these genes with those reported to be enriched in either the endocardial or coronary compartments^37^ supported a defect in coronary vessel development. 18% of the genes downregulated in mutant hearts were specific to coronary vessels while only 2% were specific to endocardial cells. Conversely, only 1% of coronary-specific genes were present in the genes upregulated in mutants while 28% were endocardial-specific (Fig. 4i). Gene set enrichment analysis (GSEA) was also used to compare the *Tie2Cre;Ino80 fl/fl* transcriptome to the endocardial/coronary data set. This analysis identified a significant enrichment of endocardial-specific genes and significant depletion of coronary-specific genes in the *Tie2Cre;Ino80 fl/fl* hearts (Fig. 4j). Thus, transcriptional analysis of whole hearts indicates a defect in coronary vessel formation.

We next analyzed whether the coronary defects persisted into postnatal stages. The coronary vasculature can compensate to repair itself at later stages when only one progenitor source is inhibited^38^. Therefore, we investigated the postnatal coronary vasculature in *Tie2Cre;Ino80 fl/fl* animals where *Ino80* would be deleted in both the SV and endocardial progenitors. At P0, neonates exhibited dramatic defects in coronary vessels, similar those observed in embryonic hearts (Supplementary Fig. 4a). Immunostaining CX40 revealed a reduction in the number of CX40^+^ arterial vessels and the persistence of CX40-negative non-compacted intermediate myocardium (Supplementary Fig. 4b). Despite a decrease in smooth muscle, the vessels that were present in mutant animals were associated with COUP-TF2^+^ pericytes (Supplementary Fig. 4c). In the animals that escaped lethality and survived to adulthood, coronary arteries were significantly smaller (Supplementary Fig. 4d, e). Therefore, vascular phenotypes persisted past the embryonic period.

### Defects in endothelial cell migration and sprouting

**Fig. 5:**
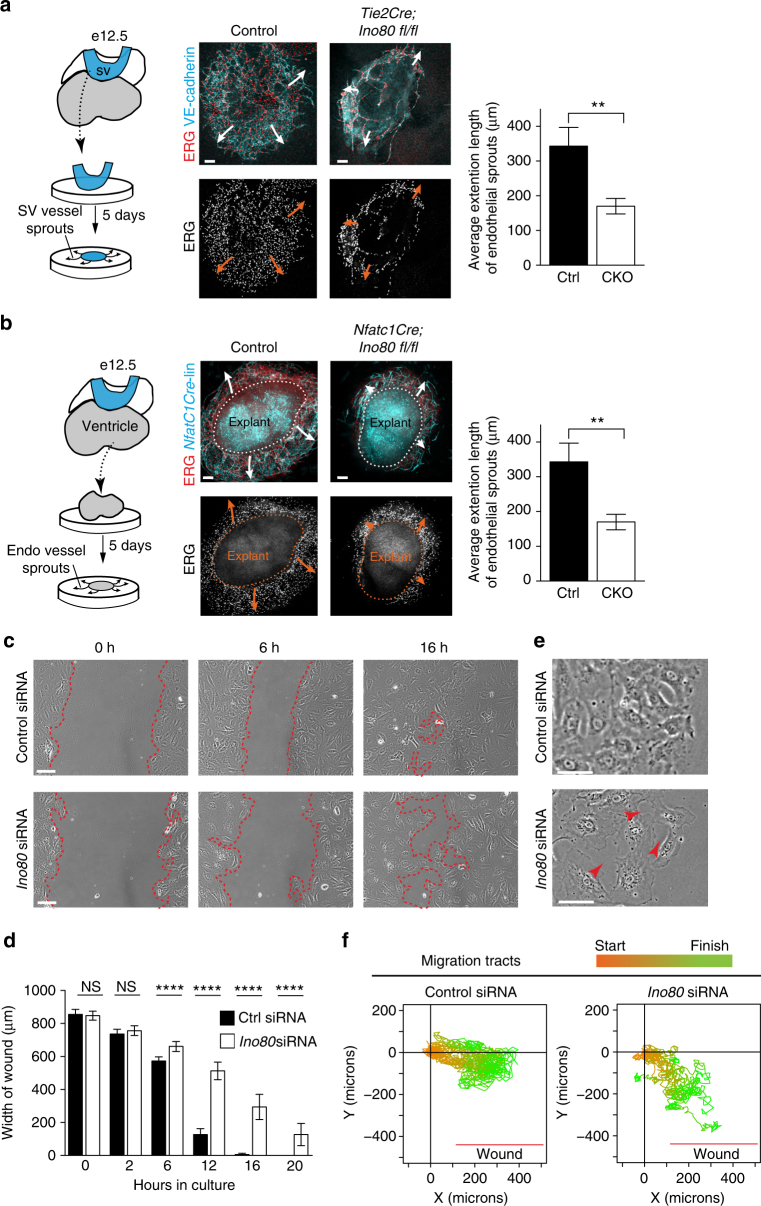
*Ino80* depletion results in faulty endothelial cell sprouting and migration. **a**, **b** Schematics, confocal images, and quantifications of sinus venosus and endocardial angiogenesis assays. Arrows indicate direction of endothelial cell sprouting. Endothelial cells are labeled with ERG and either VE-cadherin (**a**) or through *Nfatc1Cre* lineage tracing (*Nfatc1Cre*-line) (**b**). Images are representative of the following number of replicates: sinus venosus (control, *n* = 5 heart explants; mutant, *n* = 3 heart explants) (**a**) and ventricles (*Ino80* deleted using *Nfatc1Cre)*, (control, *n* = 8 heart explants; mutant, *n* = 3 heart explants). Scale bars: 100 μm. Sprouting of endothelial cells is significantly decreased in *Ino80-*deficient explants from both the sinus venosus (*Ino80* deleted using *Tie2Cre)*, (control, *n* = 5 heart explants; mutant, *n* = 3 heart explants) (**a**) and ventricles (*Ino80* deleted using *Nfatc1Cre*), (control, *n* = 8 heart explants; mutant, *n* = 3 heart explants) (**b**). (**) *P* < 0.01, evaluated by Student’s *t*-test. **c**–**f** In vitro wound assays with control or *Ino80-*depleted primary Human Umbilical Vein Endothelial Cells (HUVECs). **c** Migration to fill the wound is slower and less collective in *Ino80-*depleted cells. Images are representative of the following number of replicates: Control, *n* = 6; *Ino80* siRNA, *n* = 6. Scale bars: 100 μm. **d** Quantification of wound closure. Error bars in graphs are standard deviation. (Control, *n* = 6; *Ino80* siRNA, *n* = 6). NS nonsignificant, ****P < 0.0001, evaluated by Student’s *t*-test. **e** Representative higher magnification highlights the abnormal space (arrowheads) between mutant cells at the migration front. Scale bars: 50 μm. (Control, *n* = 6; *Ino80* siRNA, *n* = 6). **f** Migration tracts (*n* = 20 cells/condition) color-coded to highlight starting (orange) and ending (green) points show the decreased directionality of mutant cells. Error bars in graphs are standard deviation

In vitro experiments were next used to better understand the role of *Ino80* in endothelial cells. We performed a sprouting assay using explanted SVs or ventricles, the latter being a source of endocardial cells. Plating these tissues on Matrigel resulted in vessel outgrowth over a 5-day period (Supplementary Fig. 5a)^14^. As seen in vivo, endothelial/endocardial sprouting was reduced in both mutant SVs and ventricle explants when compared with controls (Fig. 5a, b). (Note that *Tie2Cre* instead of *ApjCreER* was used for SV sprouting assays to obtain the highest recombination rates since microdissection of the SV obviated the need for a specific Cre line.) We concluded that proper sprouting angiogenesis requires endothelial *Ino80*.

Since it appeared that mutant endothelial cells were not properly migrating, an assay to directly assess cell migration was used. *Ino80* was depleted in primary human umbilical endothelial cells (HUVECs) using targeted siRNAs, and time lapse videos were used to follow cell migration during experimental wound closure (i.e., scratch assays). Cells treated with control siRNAs migrated toward the cell-free region to close the wound by 16 h (Fig. 5c, d). In contrast, treating cells with *Ino80-*specific siRNA caused a significant delay in wound closure (Fig. 5c, d). In addition, *Ino80* knockdown cells had more space between individual cells (Fig. 5e), and tracing migration tracts showed that they moved in independent directions instead of as a collection in the direction of the wound (Fig. 5f and Supplementary Movies 1, 2). These data show that directional endothelial cell migration is defective in the absence of *Ino80*, and support a model where the cardiac phenotype in endothelial-specific *Ino80* knockouts derives in large part from a defect in coronary development.

### Endothelium supports heart growth independent of blood flow

**Fig. 6:**
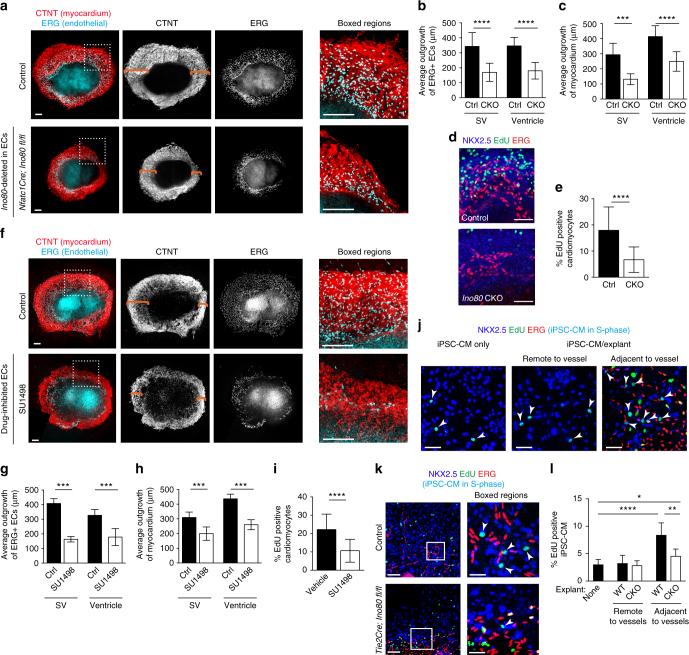
Endothelial cells support myocardial growth in the absence of blood flow in an *Ino80*-dependent manner. Heart ventricle and sinus venosus (SV) explants cultured for 5 days and immunostained for myocardium and endothelial cells. **a** Confocal images of ventricular explants show that expansion of myocardium (orange brackets) and endocardial-derived cells is decreased when *Ino80* is deleted using *Nfatc1Cre*. Images are representative of the following number of replicates: control, *n* = 8 heart explants; mutant, *n* = 5 heart explants. Scale bars: 100 μm (low and high magnification). **b**–**e** Quantifications show that endothelial sprouting (**b**), myocardial outgrowth (control, *n* = 8 heart explants; mutant, *n* = 5 heart explants) (**c**) and cardiomyocyte proliferation (control, *n* = 6 heart explants; mutant, *n* = 8 heart explants) (**d**, **e**) are significantly reduced. Images are representative of the following number of replicates: control, *n* = 6 heart explants; mutant, *n* = 8 heart explants. Scale bars: 100 μm (**d**). Error bars in graphs are standard deviation. ***P < 0.001; ****P < 0.0001, evaluated by Student’s *t*-test (**d**). **f**–**i** Inhibiting endothelial cell growth through targeting VEGFR with SU1498 recapitulates the effect of *Ino80* mutation (**f**), significantly decreasing vessels sprouting (**g**), myocardial expansion (control, *n* = 6 heart explants; SU 1498 treatment, *n* = 6 heart explants) (**h**), and cardiomyocyte proliferation (control, *n* = 10 heart explants; SU 1498 treatment, *n* = 8 heart explants) (**i**). Error bars in graphs are standard deviation. ***P < 0.001; ****P < 0.0001, evaluated by Student’s *t*-test. Scale bars: 100 μm (low and high magnification). Images are representative of the following number of replicates: control, *n* = 6 heart explants; SU 1498 treatment, *n* = 6 heart explants (**f**). **j**, **k** Images of human induced pluripotent cell-derived cardiomyocytes (iPSC-CMs) cultured either alone or with mouse ventricle explants. Proliferating NKX2.5^+^EdU^+^ iPSC-CMs are turquoise and indicated by arrowheads. Images are representative of the following number of replicates: control *n* = 10 heart explants, mutant *n* = 4 heart explants, iPSC-CM only control *n* = 7. Scale bar: 25 μm (**j**); 100 μm (**k**; low magnification) and 25 μm (**k**; high magnification). **l** Quantification of iPSC-CM proliferation within three cells lengths from endothelial cells reveals a significant increase over regions distant from vessels or iPSC-CM cultures only (control *n* = 10 heart explants; mutant *n* = 4 heart explants; iPSC-CM only control *n* = 7). This increase is significantly dampened when endothelial cells are depleted of *Ino80*. Error bars in graphs are standard deviation. *P < 0.05; **P < 0.01; ****P < 0.0001, evaluated by Student’s *t*-test. Scale bars: 100 μm

The above data suggest that coronary vessels are critical for compact myocardial growth during embryogenesis. In order to investigate a potential blood flow-independent pathway for this activity, we developed an in vitro model to assess the interactions between endothelial and myocardial cells in the absence of blood flow. Culturing either SVs or ventricles, as described in Fig. 5a, b and Supplementary Fig. 5, resulted in not only the sprouting of endothelial cells, but also the expansion of myocardium (Fig. 6a). In SV and ventricle cultures from various endothelial-specific *Ino80* deleted animals, defective endothelial sprouting was accompanied by decreased myocardial expansion (Fig. 6a–c). Importantly, explants were obtained at e12.5 when there was no difference in mutant heart size (Fig. 1b–d) and tissues were the same size on day 1, making these cultures a comparable measure of in vitro growth (Supplementary Fig. 5). Assessing cell proliferation by incubating cultures with EdU and immunostaining with NKX2.5^+^ showed that cardiomyocyte proliferation was also decreased (Fig. 6d, e).

If the decreased cardiomyocyte expansion/proliferation resulted from defective angiogenesis, we should be able to recapitulate the in vitro result by directly inhibiting endothelial growth. Wild-type cultures were treated with the VEGFR2 inhibitor, SU1498, to directly inhibit endothelial cell proliferation and survival (Fig. 6f). Treatment of SV and ventricular cultures decreased vessel sprouting (Fig. 6f, g), and this was coincident with a decrease in myocardial expansion and proliferation (Fig. 6f, h, and i). These data support the hypothesis that endothelial cells stimulate myocardial expansion in the absence of oxygenated blood flow, and that *Ino80-*deficient cells are incapable of properly carrying out this function.

The above experiments show that endothelial cells support cardiomyocyte proliferation, leading us to conclude that the non-compaction phenotype in *Ino80-*deficient mice could result from a decrease in coronary vessels. However, an additional possibility is that Ino80 stimulates the production of endothelial-derived factors important for compaction. To begin addressing this, we co-cultured control and mutant ventricle explants with induced pluripotent stem cell-derived cardiomyocytes (iPSC-CMs). The explants also contained the *Rosa*^*mTmG*^ allele so that NKX2.5^+^ iPSC-CMs could be distinguished from murine cells because they lacked red fluorescence. Co-cultures were maintained for 9 days to allow vessels to leave the explants and migrate onto the iPSC-CM layer. Performing an EdU labeling assay revealed that iPSC-CM proliferation rates were increased in cells directly adjacent to sprouting vessels (i.e., within three cell lengths from endothelial cells), indicating a short-range paracrine effect (Fig. 6j, l). Thus, endothelial cells and/or endothelial-associated cells that migrate onto the iPSC-CM layer increase proliferation in neighboring human cardiomyocytes.

Since the increase in iPSC-CMs proliferation was very localized, restricting the analysis to cells directly adjacent to sprouting vessels should allow us to assess whether *Ino80*-deficient endothelial cells stimulate cardiomyocyte proliferation. We found that iPSC-CMs in regions with comparable numbers of endothelial cells were less proliferative when explants were derived from *Tie2Cre;Ino80 fl/fl* animals (Fig. 6k, l). These data suggest that not only is *Ino80* important for vessels growth, but also in allowing endothelial cells to stimulate cardiomyocyte proliferation (either through direct signaling or indirectly through another cell type in the culture).

### Ino80 inhibits E2F target gene expression

**Fig. 7:**
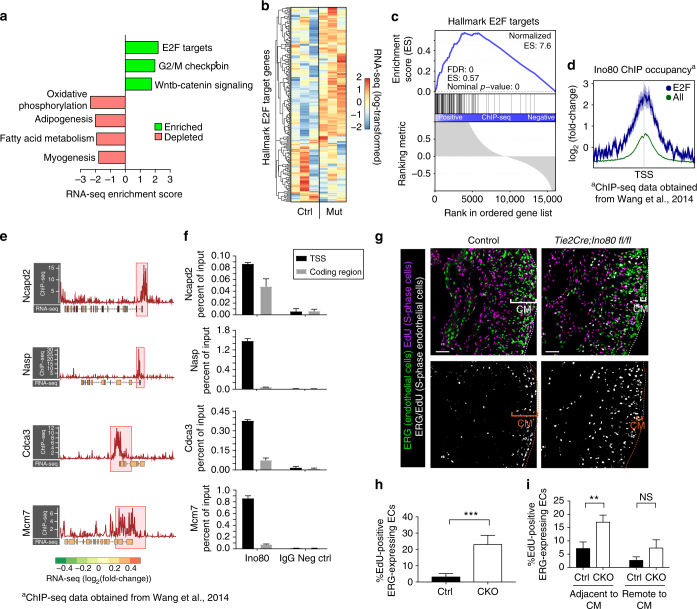
Deletion of Ino80 increases E2F-regulated gene expression and endothelial S-phase progression. **a** GSEA of transcriptional changes in *Tie2Cre;Ino80 fl/fl* hearts compared to control hearts. All significantly enriched hallmark gene sets are shown with corresponding normalized enrichment scores (ES). **b** Heatmap of hallmark E2F target gene expression in each of three control (ctrl) and three *Tie2Cre;Ino80 fl/fl* mutant (mut) e13.5 hearts. Each column represents RNA-seq from an individual heart. Color represents regularized-log-transformed counts after scaling by row (i.e., gene). (Control *n* = (**c**) GSEA analysis of Ino80 ChIP occupancy in hallmark E2F target gene promoters in J1 ESCs. **d** Composite profiles of Ino80 occupancy at hallmark E2F target gene promoters (blue line) compared to the genome-wide average (green line). **e** Ino80 ChIP occupancy and corresponding RNA-seq expression changes at individual E2F-dependent loci. ChIP-seq signals (red line, -log_10_-pval) and promoters (red boxes, 1.5 kb upstream and 2 kb downstream of TSS) are shown. Mutant RNA-seq data (log_2_[fold change]) is represented by color of exon. **f** Ino80 ChIP enrichment at transcriptional start sites (TSSs) and coding regions for indicated genes in HUVECs. Rabbit IgG is shown as a negative control. ChIP-qPCR results are shown as percent of input. Standard deviation was calculated from three technical replicates. **g** EdU incorporation assays show that endothelial cells have an increased number of cells in S-phase. Top panels show immunofluorescence to label endothelial cells (ERG) and EdU positivity (green). Bottom panels show overlap between the two signals. Images are representative of the following number of replicates: control, *n* = 4 hearts, mutant, *n* = 3 hearts. Scale bars: 100 μm. **h**,** i** Quantification of EdU-positive endothelial cells show there is a significant 10-fold increase at e15.5 (control, *n* = 4 hearts; mutant, *n* = 3 hearts) (**h**) and at e12.5 (control, *n* = 3 hearts; mutant, *n* = 3 hearts) (**i**) in cells adjacent to the compact myocardium. Error bars in graphs are standard deviation. NS nonsignificant, **P < 0.01; ****P < 0.0001, evaluated by Student’s *t*-test. CM compact myocardium

To understand how *Ino80* regulates the genome to influence angiogenesis, we analyzed gene expression and Ino80 ChIP data. The RNA-sequencing data from e13.5 control and *Tie2Cre;Ino80 fl/fl* hearts was further compared to hallmark gene sets in the Molecular Signatures Database^39^, each of which represent an essential compilation of genes that convey a specific biological state or function. This analysis identified the enrichment of proliferative pathways, including “E2F Targets” and “G2/M Checkpoint”, in *Tie2Cre;Ino80 fl/fl* hearts (Fig. 7a, Supplementary Fig. 6a). Both these gene sets contain 200 genes from 420 founder data sets that are coherently related to cell cycle progression and proliferation^39^. Indeed, many cell cycle regulated genes, such as the MCM replication licensing factors, are upregulated in the *Tie2Cre;Ino80 fl/fl* hearts compared to controls (Fig. 7b). Conversely, “Oxidative Phosphorylation” and “Myogenesis” gene sets are decreased in *Ino80* mutant hearts compared to control, which likely reflects a relative depletion of mature, mitochondria-rich cardiomyocytes as the non-compaction phenotype emerges (Fig. 7a). These data suggest the *Ino80* regulates heart development by affecting E2F target gene expression.

In order to explore whether Ino80 directly or indirectly regulates E2F targeted genes, we analyzed previously published Ino80 chromatin immunoprecipitation-sequencing (ChIP-seq) data procured from mouse embryonic stem cells (ESCs)^30^. This data set revealed that Ino80 preferentially binds to transcriptional start sites (TSS) in a subset of genes in the mammalian genome (Supplementary Fig. 6b). GSEA analysis demonstrated that Ino80 occupancy was significantly enriched at E2F-regulated promoters (Fig. 7c). Composite plots of E2F-regulated promoters also showed an enrichment of Ino80 occupancy compared to all promoters (Fig. 7d). Statistical analysis showed that Ino80 occupancy correlates with suppression of E2F-mediated transcription (Supplementary Fig. 6c). Computational clustering of these promoters based on Ino80 occupancy and position demonstrates that E2F-regulated genes with relatively high Ino80 occupancy either 3′ or 5′ of the transcriptional start site (C3, C4) have elevated expression compared to promoters with lower occupancy and/or dispersed promoter positioning (C1, C2) (Supplementary Fig. 6c). Thus, both abundance and positioning of Ino80 are correlated with the degree of transcriptional repression. The enrichment of Ino80 at the TSSs of specific E2F-regulated genes in ESCs is shown in Fig. 7e, which is confirmed in HUVECs and demonstrates Ino80 regulation of E2F genes specifically in vascular endothelial cells (Fig. 7f). Collectively, the expression data and promoter occupancy suggest that Ino80 normally functions to repress E2F target genes in the developing heart.

E2F family transcription factors play diverse roles in facilitating or inhibiting cell cycle progression^40^, thus we investigated which of these behaviors was affected in *Tie2Cre;Ino80* mutant hearts. Specifically, hearts from EdU-treated control and mutant embryos (e15.5) were analyzed to assess the percentage of cells in S-phase. Compared with controls, the percentage of EdU-positive endothelial cells was ~10-fold higher in mutant hearts (Fig. 7g, h). We also investigated endothelial EdU labeling at a developmental time point when endocardial cells are migrating into the myocardium to form coronary vessels (e12.5). Interestingly, the increase in EdU positivity was in endocardial cells adjacent to the forming compact myocardium where these cells enter ventricular tissue and where angiogenic signals would presumably be located (Fig. 7i)^14^. These data support the model that Ino80 functions to suppress endothelial cell cycle gene expression during development, which could be important to promote angiogenesis in growing tissues.

## Discussion

**Fig. 8:**
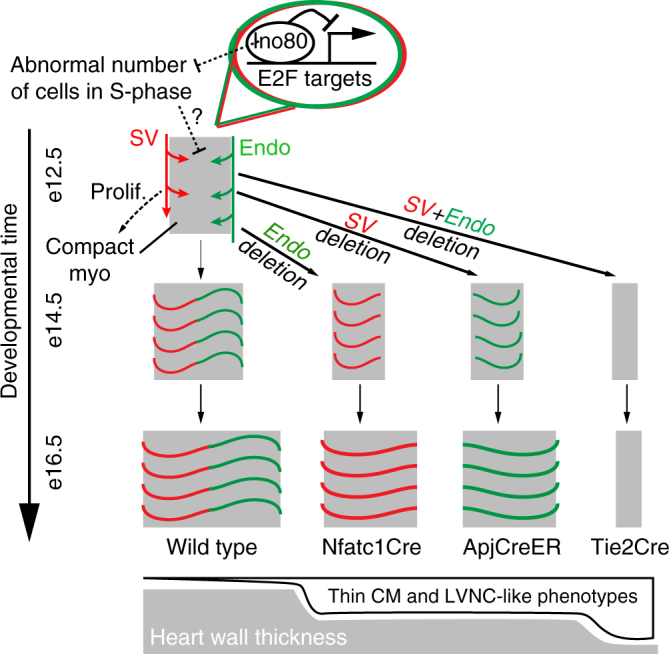
Model for the role of Ino80 and regional coronary vascularization during heart wall growth. Two major progenitors of coronary vessels exist, the sinus venosus (SV, red) and endocardial cells (endo, green). These progenitors grow into the ventricle walls from different sides of the heart and connect to form the coronary vascular bed, which is coincident with expansion of the compact myocardium. Deletion of *Ino80* from each progenitor pool separately, or together using cell type specific Cre lines, results in either medium or severe heart wall thinning/ventricular non-compaction, respectively. Ino80 occupies E2F target gene promoters and suppresses their expression, limiting cells in S-phase and allowing proper angiogenesis. These models highlight that increasing the amount of vasculature through access to multiple progenitor sources accelerate expansion of the heart wall during development. Major depletion of coronary vasculature results in severe LVNC-like phenotypes

In this study, we investigated the role of the Ino80 chromatin remodeler during cardiac development (Fig. 8). Collectively, our data demonstrate that Ino80 normally functions to suppress E2F-mediated gene expression in cardiac endothelial cells and supports productive coronary angiogenesis. Coronary vessels from both the SV and endocardial progenitor pools are needed to ensure proper myocardial compaction and heart wall expansion. Furthermore, compact myocardial growth is promoted by endothelial cells in the absence of blood flow in an *Ino80*-dependent manner. Absence of these developmental processes lead to congenital heart defect phenotypes.

Our data also show that the process of blood vessel angiogenesis was particularly sensitive to deletion of *Ino80*. For example, there were no apparent defects in endothelial-specific knockout hearts prior to coronary vessel development or when the gene was deleted in other cardiac cell types. Endothelial-specific deletion of *Ino80* severely stunted coronary angiogenesis, which correlated with a dramatic decrease in the size of the compact heart wall and a phenotype reminiscent of the human congenital heart disease, LVNC. *Ino80* deletion in either the SV-derived or endocardial-derived coronary vessels resulted in an intermediate phenotype, indicating that possession of both vessel sources allows the rapid expansion of the heart wall that occurs during mid-gestation. Notably, in these more mild cases, the phenotype was always more prominent in the apex region. The underlying reason is not known, but we hypothesize that it relates to the fact that the apex is the last region of the heart wall to compact and one of the last regions to receive full coronary vascularization. Our additional observation that endothelial cells stimulate cardiomyocyte proliferation in an in vitro model suggests that coronary vessels support myocardial expansion via blood flow-independent means, possibly through a currently unidentified angiocrine signal. Collectively, our model reveals that providing two sources of blood vessels during development functions to accelerate heart wall expansion.

Bioinformatic and in vivo analyses uncovered potential mechanistic roles for the Ino80 remodeler in the regulation of embryonic development. Analysis of Ino80 ChIP data produced in murine ESCs revealed that Ino80^30^ preferentially bound to E2F-regulated promoters, and expression of these genes were increased in mutant hearts. These data suggest that Ino80 functions to suppress E2F transcriptional activity. Other remodeler families with links to congenital heart disease phenotypes in human disease and/or mouse models have also been implicated in E2F gene regulation^41–44^. It will be interesting to investigate whether E2F deregulation is a general feature of the etiology of congenital heart defects resulting from mutations in chromatin remodelers.

The E2F family of transcription factors have well characterized roles in the regulation of the cell cycle in response to external proliferative cues^40^. E2F1–3 are primarily categorized as transcriptional activators, while E2F4-8 are primarily or exclusively repressors. E2F-mediated transactivation is specifically required for progression to S-phase. Conversely, inhibitory E2Fs recruit repressive factors such as retinoblastoma (Rb)-family pocket proteins to the promoters of cell cycle genes, inducing G1 arrest and entry into G0^40, 45^. In fact, deletion of the retinoblastoma (Rb) family proteins results in various congenital cardiac defects, including hyperplasia, increased heart size, and double outlet right ventricle (DORV)^46, 47^. These animals also exhibit an endothelial-specific increase in proliferation^47^. Thus, E2F function is critical for restraining endothelial cell proliferation during normal heart development and preventing congenital heart defects.

In vivo, we found an increase in the number of endothelial cells in S-phase in the *Ino80* knockout hearts, particularly in regions where coronary vessels emerge. Thus, we propose that Ino80 functions to buffer against abnormally high S-phase occupancy in the presence of growth factors so that morphogenic processes such as migration can accompany tissue expansion. Indeed, computational modeling of angiogenesis has concluded that this process is severely impaired when proliferation rates are too high^48^. The modeling indicates that there must be a balance between cell proliferation and migration for endothelial cell-lined vessels to fill available tissue space.

The importance of balancing endothelial cell activation can be appreciated during manipulations of the Notch pathway. Notch signaling is well known for its role in inhibiting proliferation and counteracting the over activation of endothelial cells. Notch mutations result in overly dense vessel networks that do not undergo angiogenesis properly, which is particularly obvious in the retina where their migration is severely stunted^49–51^. We did not detect statistically significant changes in the Notch pathway in our *Ino80* RNA-seq data sets, either computationally or experimentally. However, similar to Notch mutants, Ino80-deficient vessels are also overly dense and fail to properly migrate onto their target tissue. We propose that abnormal S-phase occupancy and/or proliferation inhibits the ability of Ino80-deficient coronary endothelial cells to properly migrate outward and cover the heart, where the vessels would normally stimulate compact myocardium proliferation. Therefore, our data suggest that buffering cell proliferation via chromatin remodeler-mediated transcriptional regulation is another important aspect of productive angiogenesis.

In addition to increasing our understanding of cardiac angiogenesis, our data show that human congenital heart disease phenotypes can arise from defective coronary vascularization. Other mouse models of LVNC also exist, which include mutations with cell autonomous roles in both cardiomyocytes and endothelial cells, which aligns with the observation that human LVNC is associated with mutations in both cardiomyocyte and endothelial genes^1^. In summary, we used specific Cre lines to show that defects in coronary angiogenesis leads to ventricular non-compaction in mice, further supporting the possibility that defective angiogenesis also underlies LVNC in some human patients.

## Methods

### Mouse lines

Stanford complies with all federal and state regulations governing the humane care and use of laboratory animals, including the USDA Animal Welfare Act and our Assurance of Compliance with PHS Policy on Humane Care and Use of Laboratory Animals. The laboratory animal care program at Stanford is accredited by the Association for the Assessment and Accreditation of Laboratory Animal Care International (AAALAC Int’l). Experiments were conducted with NIH and Stanford University policies governing animal use and welfare. The following strains used were: wild-type (CD1 and FVB, Charles River Laboratories) and *Myh6Cre* (The Jackson Laboratories Stock number 011038, B6.FVB-Tg(Myh6-cre)2182Mds/J), *Tie2Cre* (The Jackson Laboratories Stock number 004128, B6.Cg-Tg(Tek-cre)12Flv/J), *Nfatc1Cre*^29, 30^, *ApjCreER*^18^, and *Ino80 flox*. The *Ino80* mouse line (Ino80^tm1a(EUCOMM)Hugu^) was generated by the International Knockout Mouse Consortium. The tm1a allele (http://www.mousephenotype.org/data/alleles/MGI:1915392/tm1a(EUCOMM)Hmgu) was crossed to mice with widespread Flp recombinase expression (The Jackson Laboratory Stock number 019100, B6N.Cg-Tg(ACTFLPe)9205Dym/CjDswJ) to create a conditional knockout allele of *Ino80* where exon 6 is flanked by *loxP* sites. The following genotyping primers were used to distinguish between wild-type and *Ino80 flox* alleles: (Forward: 5′-ACCTGCTGGCACCTTTCCAGTCT-3′, Reverse: 5′-CCACTACACACAGCAGATACACAT−3′). For experiments, Cre-expressing *Ino80 flox*/+ male mice were crossed with *Ino80 fl/fl* females. In some cases, these animals also possessed the *ROSAmTmG* Cre reporter allele (The Jackson Laboratories Stock number 007676, B6.129(Cg)-Gt(ROSA)26Sortm4(ACTB-tdTomato,-EGFP)Luo/J). Transgenic mice were on mixed backgrounds and ages (embryonic and adult) are indicated with each experiment. Both male and female embryos (1:1 ratio) were included in analyses as we did not genotype for gender.

When using the *ApjCreER* to excise the *Ino80*, pregnant females were given 4-OHT (H6278 Sigma) by oral gavage at e10.5 and 11.5.

### Mouse embryo fibroblast isolation and western analysis

MEFs were prepared from e13.5 control and *Tie2Cre;Ino80 fl/fl* embryos. Cells were grown in DMEM with 10% FBS and infected with either adenovirus expressing Cre recombinase (Ad5-CMV-Cre-GFP) or empty vector (Ad5-CMV-GFP) after 4 passages. After 48 h, GFP expression was measured at >95%. After 72 h after infection, protein was collected for western analysis using anti-Ino80 antibody (Abcam ab105451) and anti-tubulin antibody (Millipore MAB1864). Uncropped western images shown in Supplemental Fig. 7.

### Isolation of heart endothelial cells

Hearts from *Tie2Cre;Ino80 fl/fl* and *Tie2Cre;Ino80 fl/+* embryos were collected at e15.5, minced with a sterilized razor blade, and incubated in 0.5 ml dissociation solution (collagenase, dispase, and DNase) at 37 °C for 20 min. Once digested, 10 ml of PBS with 2% bovine calf serum was added and the digested heart tissues were filtered through 40 µm cell strainer. After centrifugation, cell pellets were treated with red blood cell lysing solution for 5 min, and PBS with 2% bovine calf serum were added. After centrifugation, cell pellets were resuspended in PBS with 2% bovine calf serum, blocked with rat IgG for 10 min, and stained with rat anti-mouse CD31 APC (eBioscience, 17-0311-80, 1:500) and anti-mouse CD45 (Biolegend, 103101, 1:200). Viable CD45-negative, CD31-positive cells were sorted on BD FACS Aria and collected into 0.5 ml of Trizol LS reagent (Invitrogen, 10296028) directly for RNA extraction. To analyze *Ino80* and *Vegfr2* mRNA in sorted populations from e14.5 hearts cells were sorted using antibody-coupled magnetic beads, Purified Rat Anti-Mouse CD144 (BD Pharmaceutical, 550548) and Goat Anti-Rat IgG Magnetic Beads (NEB, S1433S).). Total RNA was extracted according to the manufacturer’s instructions (Qiagen, 74034) and converted to cDNA using iScript RT Supermix (BIO-RAD, 1708840). The SYBR Green qPCR master mix (BIO-RAD, 1725121) was used and quantitative RT–PCR was performed on Real-Time PCR System (Bio-Rad, CFX96 Touch Real-Time PCR dectection system). *Ino80* Primers: Forward: 5′-CAGGCCTGCTCTCTACATACG-3′, Reverse: 5′-TGTTAACCACCACTCCTCCAC-3′, Vegfr2 Primers: Forward: 5′-CTCTGTCAAGTGGCGGTAAA-3′, Reverse: 5′-TCAGGAAGCCACAAAGCTAAA-3′, Gapdh: Forward: 5′-GTGGCAAAGTGGAGATTGTTG-3′, Reverse: 5′-CGTTGAATTTGCCGTGAGTG-3′.

### Histology, in situ hybridization, and immunohistochemistry

Embryos from timed pregnancies (morning of plug designated e0.5) were isolated in PBS and then fix overnight in 4% paraformaldehyde (PFA) overnight at 4 °C. The following day, embryos were washed in PBT (PBS containing 0.1% Tween-20), dehydrated in an ascending methanol sequence, xylene treated, embedded in paraffin, and sectioned at 7 µm. Hematoxylin and eosin (H&E) staining was performed on deparaffinized slides as reported.

In situ hybridization on paraffin section was performed as described previously^10^. Dissected embryos were fixed in 4% PFA in diethyl pyrocarbonate (DEPC)-treated PBS at 4 °C overnight, washed in PBS, dehydrated in methanol series, cleared in xylene and embedded in paraffin. Embryos were sectioned at 10 um thickness. Slides were pre-incubated in hybridization buffer at 65 °C for 2 h then incubated with 1 ug/ml probes in hybridization buffer at 65 °C overnight. Antisense *Cx40*, *Hey2*, *N-myc*, and *Tbx20* probes were labeled with digoxigenin (DIG)-UTP using the Roche DIG RNA labeling System according to the manufacturer’s guidelines. The *Cx40*, *Hey2*, *N-myc*, and *Tbx20* plasmid were kind gift from Dr. José Luis de la Pompa. After washing in salt sodium citrate (SSC) buffer, slides were incubated with alkaline phosphatase-conjugated anti-digoxigenin-alkaline phosphatase antibody (Roche, 11093274910) at 4 °C overnight and signal was visualized with BM purple alkaline phosphatase substrate (Roche, 1144207001). Slides were mounted using entellan mountain medium (Electron Microscopy Sciences, 14800) and imaged using Zeiss Axioimager A2.

Immunofluorescence was performed on 7 µm deparaffinized sections. Briefly, sections were subjected to antigen retrieval in *Tris* buffer pH 10.2 for 10 min, washed in 0.1% PBT and incubated in blocking buffer (0.5% milk powder, 99.5% PBT) for 2 h at room temperature. Primary antibodies were incubated in blocking buffer overnight at 4 °C. The following day, the sections were washed three times with PBT and incubated for 1 h with corresponding secondary antibodies in blocking buffer at room temperature. After three washes in PBT, DAPI (Sigma-Aldrich, 1:2000) was added to counter-stain the nuclei. The sections were mounted using Prolong Gold Antifade Reagent (Invitrogen, P36934) and imaged using either Zeiss Axioimager A2 or Zeiss LSM-700 confocal miscroscope. The following primary antibodies were used: Aquaporin1 (Temecula, AB2219, 1:1000), ERG (Abcam, ab92513, 1:1000), ENDOMUCIN (Santa Cruz, sc-65495, 1:250), hPROX1 (R&D Systems, AF2727, 1:250), COUP-TF2 (Perseus Proteomics, PP-H7147-00, 1:1000) and CX40 (Alpha Diagnostic Intl. Inc., CX40-A, 1:1000). Secondary antibodies were Alexa Fluor conjugates 488, 555, and 647 (Life Technologies) at 1:500. The antibodies used for immunostaining were listed in Supplementary Table 1.

### Immunofluorescence on frozen tissue sections

Embryos at different stages were collected and fixed in 4% PFA for 1 h at 4 °C. Hearts were collected, embedded in Optimal Cutting Temperature (O.C.T.) compound, sectioned (20 μM), and stained with antibodies. Immunofluorescence staining was performed on microscope slides. Primary antibodies were diluted in blocking solution (5% goat serum, 0.5% Triton X-100 in PBS) and incubated overnight at 4 °C. Tissues were washed with 0.5% PBT (PBS containing 0.5% Tween-20) for 30 min three times followed by a 3 h room temperature incubation in secondary antibodies (diluted in goat serum). These tissues were washed again as previously described. Using the staining system from Vector Laboratories, the specimens were placed in Vectashield and imaged using an inverted Zeiss LSM-700 confocal microscope. Captured images were digitally processed using Zen (Carl Zeiss), ImageJ (NIH), Photoshop (Adobe Systems) and Illustrator (Adobe Systems). The primary antibodies used in the immunofluorescence analyses were: VE-cadherin (BD Biosciences, 550548, 1:125), Myomesin (Developmental Studies Hybridoma Bank, mMaC myomesin B4, 1:500) and CTNT antibody (Developmental Studies Hybridoma Bank, CT3-c, 1:500). Secondary antibodies were Alexa Fluor conjugates 488, 555, and 647 (Life Technologies) at 1:250. DAPI was used to label nuclei (Sigma-Aldrich, 1:2000).

### Whole-mount immunofluorescence staining

Whole-mount immunostaining was performed as previously described^18, 52^. Antibodies used were: VE-cadherin (BD Biosciences, 550548, 1:250), VEGFR2 (R&D Systems, AF644; 1:250), DACH1 (Proteintech, 10914-1-AP, 1:1000), Myosin (Smooth Muscle) Heavy Chain (Alfa Aesar, BT 562, 1:1000), CTNT (DSHB, CT3-3, 1:1000) and NKX2.5 (Santa Cruz, sc-8697, 1:250). Secondary antibodies were Alexa Fluor conjugates (488, 555, 647, Life Technologies; 1:250).

### Explant culture, immunostaining and quantification

Sinus venosus explants or whole ventricles were dissected from e12.25 embryos. Samples were rinsed with cold PBS to remove blood cells and placed in the Matrigel (1:200 BD Biosciences) with culture media (EGM-2 MV, Clonetics, CC-4147) in 24-well plates (Costar, 3524). Explants were cultured in 5% O_2_, 5% CO_2_ at 37 °C for 5 days before samples were fixed with 4% PFA in PBS for 15 min. After fixation, explants were washed three times with 0.5% PBT and immunofluorescence staining was performed directly within the 24-well culture plates. Explants were incubated with primary antibodies in 0.5% PBT overnight at 4 °C. Explants were washed with 0.5% PBT six times for 6 h and then incubated with corresponding secondary antibodies in 0.5% PBT overnight at 4 °C. The day after, explants were washed three times and nuclei were counter-stained with DAPI. After three washes in PBS, explants were placed in PBS and imaged using an inverted Zeiss LSM-700 confocal microscope. Captured images were digitally processed using ImageJ (NIH) and Photoshop (Adobe Systems). Antibodies used were: VE-cadherin (BD Biosciences, 550548, 1:250), ERG (Abcam, ab92513, 1:1000), CTNT (DSHB, CT3-3, 1:1000), NKX2.5 (Santa Cruz, sc-8697, 1:250). Secondary antibodies were Alexa Fluor conjugates (488, 555, 647, Life Technologies; 1:500). Small-molecule inhibition was performed by the addition of SU1498 (20 µM; Calbiochem) dissolved in DMSO directly to culture media after 2 days culture. An equal concentration of DMSO was added to the control media.

To quantify outgrowth of endothelial cells and expansion of myocardium, explants were immunostained with ERG (endothelial cells) and CTNT (cardiomyocytes). The distance of vessel growth from inside line of ERG-positive cells in the compact myocardium (ventricle culture) or the center of the SV area (SV culture) to the maximum distance reached by the endothelial sprouts was measured in three fields per sample and averaged. For quantification of myocardial expansion, the width covered by CTNT-positive cell was measured at three different points and averaged.

### Ex vivo co-culture of human iPSC-derived cardiomyocytes and mouse embryonic heart

Human induced pluripotent stem cells (iPSCs) from a female subject with no reported cardiovascular complications were obtained from the Stanford Cardiovascular Institute iPSC Biobank (consent received through IRB protocol #29904). iPSCs are cultured in E8 media (Gibco) for maintenance of pluripotency state. At 95% confluency, iPSCs were subjected to chemically defined cardiomyocyte differentiation protocol^53^. In brief, iPSCs were given 8 µM CHIR99021 (Selleckchem) in RPMI+B27 without insulin (Gibco) from day 0 to 2. Media was changed to RPMI+B27 without insulin from day 2 to 3, then the cells were treated with 5 µM IWR (Selleckchem) in RPMI+B27 without insulin from day 3 to 5. Media was changed to RPMI+B27 without insulin from day 5 to 7. Robust beating of cardiomyocytes was observed starting at day 8 of differentiation. From day 7 and forward, RPMI+B27 with insulin (Gibco) media were given to iPSC-derived cardiomyocytes (iPSC-CMs). Glucose starvation from day 11 to 13 was used to eliminate non-cardiomyocyte cells. At day 15, iPSC-CMs were transferred to Matrigel-coated chamber slides or 24-well plates, on which isolated mouse embryonic hearts dissected at e12.25 were placed. These co-cultures were maintained in a 1:1 mixture of RPMI+B27 with Insulin and EGM-2 MV (Clonetics, CC-4147), and cultured at 5% O_2_, 5% CO_2_ at 37 °C for 9 days. Preparation for immunostaining was performed as described above.

### Quantification of compact myocardium thickness and trabecular length

To visualize the structure of ventricles, immunostaining was performed on paraffin sections with anti-Endomucin for endocardial cells, anti-ERG for endothelial cells and anti-CTNT for cardiomyocytes. ImageJ software was used to measure the thickness of the compact myocardium (CM) and the length of trabecular myocardium (TM) in tissue sections from equivalent coronal planes of the heart. For each parameter, six measurements were taken along the lateral sides of the heart and averaged individually for both the left and right ventricle.

### Proliferation assays

Cell proliferation in vivo and in explants was quantified by detection of EdU incorporation. For in vivo proliferation rate, 50 µg/g of body weight of EdU was injected into pregnant mice intraperitoneally 3 h before embryo collection. Proliferating cardiomyocytes and endothelial cells were calculated from paraffin tissue sections as the percentage of PROX1-positive cardiomyocytes or ERG-positive endothelial cells labeled EdU in a 200 µm^2^ field of view. For explant cultures, 200 ng of EdU was added directly into 0.5 ml of media 30 min before fixing tissues. EdU-positive cells were detected with the Click-iT EdU kit (Invitrogen, C10338) according to manufacturer’s instruction. Briefly, Click-iT reaction cocktails were incubated for 30 min after the secondary antibody incubation of the immunostaining protocol (see above). Myocyte proliferation was calculated as the percentage of NKX2.5+ cardiomyocytes also labeled with EdU in a 200 µm^2^ field of view.

### Migration assay

Human Umbilical Vein Cells (HUVEC, Lonza C2517A) were grown in Endothelial Cell Growth Medium (EGM-2, Lonza) and transfected with either siRNA targeting Ino80 (Sigma-Aldrich, cat. No. EHU069661) or negative control siRNA (Ambion, cat. No. AM4636) with a final concentration of 13.8 uM. After 48 h, the growth area was scratched and cells were imaged every 15 min for 24 h in three different fields using a wide-field Zeiss Axiovert 200 M Microscope equipped with a temperature controlled CO_2_ incubation system. The migratory track of each cell was measured using the MTrackJ tool from ImageJ.

### RNA-sequencing

E13.5 embryos were dissected from *Tie2Cre;Ino80 fl/fl* pregnant females. RNA from whole hearts were used to prepare sequencing libraries with NEB Next Ultra RNA library prep kit and sequenced with 76 bp Illumina paired-end reads. Raw sequencing library quality was assessed using FastQC. Reads were then mapped to genome GRCm38 (mm10) using STAR (v2.4.2a) with gene annotations from the GENCODE primary assembly (vM6). Reads aligning to transcripts were counted using the *summarizeOverlaps* function from the GenomicAlignments R package^54^. An average of 33.9 M reads were counted for each WT replicate, vs. an average of 53.5 M reads for each KO replicate. Count data were input into DESeq2, and data were regularized-log-transformed prior to visualization by heatmap^55^. Significance results and log_2_(fold change) values were generated using the DESeq2 algorithm on untransformed count data.

Gene ranks for pre-ranked GSEA were defined as -log_10_(padj) for upregulated genes in the mutant (log_2_(fold change)>0) and (−1) × −log_10_(padj) for downregulated genes, where padj is the Benjamini-Hochberg corrected *p*-value from DESeq2. This is analogous to the ranking system used in reference^56^. Enrichment of 15597 genes with non-zero expression was evaluated within 7057 hallmark gene sets^57^ and Zhang et al.^37^ using the “weighted” enrichment statistic.

### ChIP analysis

Ino80 J1 ESC ChIP-seq data were obtained from GEO accession GSE49137 at NCBI repository^29, 30^. Specifically, SRA-formatted raw reads were programmatically downloaded with Aspera ascp executable (v.3.5.6) and converted to Fastq with fastq-dump (v.2.5.7). ChIP-seq coverage tracks were generated from raw sequencing reads using the AQUAS TF and histone ChIP-seq pipeline (https://github.com/kundajelab/chipseq_pipeline)^58^. Briefly, reads were aligned to *Mus musculus* genome assembly mm10 (for consistency with RNA-seq) using BWA, de-duplicated, converted to tagAlign format, replicate-merged, and input into MACS2.0 for fold change and *p*-value signal tracks^58, 59^. Resulting BigWig files were used for all data visualization; heatmaps and profiles were generated using SeqPlots^60^. In order to rank genes by Ino80 occupancy for GSEA analysis, promoters were first defined as the window from 1500 bp upstream of the TSS to 2000 bp downstream of the TSS, based on an initial analysis of the Ino80 ChIP signal in a 20 kb window surrounding TSSs. Reads aligning within these promoter windows were counted using the summarizeOverlaps() function from the GenomicAlignments R package, specifying mode = “Union” and ignore.strand = TRUE^1^. Count data were input into DESeq2, and log2(fold change) values from promoter IP/input counts were used in pre-ranked GSEA^55, 61^ with the “classic” enrichment statistic strategy.

Ino80 ChIP-qPCR was performed as previously described^62^. Briefly, 5 × 10e6 confluent primary HUVEC cells were fixed with 1% formaldehyde for 10 mins. and sonicated using a Diagenode BioRuptor to average fragment length of 300–500 bp. Immunopreciptation was performed either 10 ug of anti-Ino80 antibody (Abcam, ab105451) or rabbit IgG (negative control) overnight. Crosslinking was reversed by heating at 65 °C overnight and purified DNA was used in quantitative PCR (qPCR).

### Data availability

RNA-sequencing data that support the findings of this study are under accession number GSE98082 in GEO repository. The rest of the data is available from the authors upon reasonable request.

### Statistical analysis

Statistical analyses were performed using Prism (Graphpad). Data are represented as mean ± sd. For animal knockout studies, no statistical methods were used to predetermine sample size; sample size was determined based on mouse genetics. Crosses were performed until a minimum of 3–10 experimental animals (i.e., mutants) from multiple litters were obtained. No randomization or blinding was performed. Litter mate controls were always used for analyzing experimental animals. Unpaired *t*-test (two-tailed) were performed to assess statistical significance between two sample groups. A *p* < 0.05 was considered statistically significant.

## Electronic supplementary material

Supplementary Information

Description of Additional Supplementary Files

Supplementary Movie 1

Supplementary Movie 2
