# Supplementary material for: Endothelial deletion of Ino80 disrupts coronary angiogenesis and causes congenital heart disease
Source: Nat Commun. 2018 Jan 25;9:368. doi: 10.1038/s41467-017-02796-3 (PMC5785521; doi:10.1038/s41467-017-02796-3)
Supplement: Supplementary file 1 — Supplementary Information [file 41467_2017_2796_MOESM1_ESM.pdf]

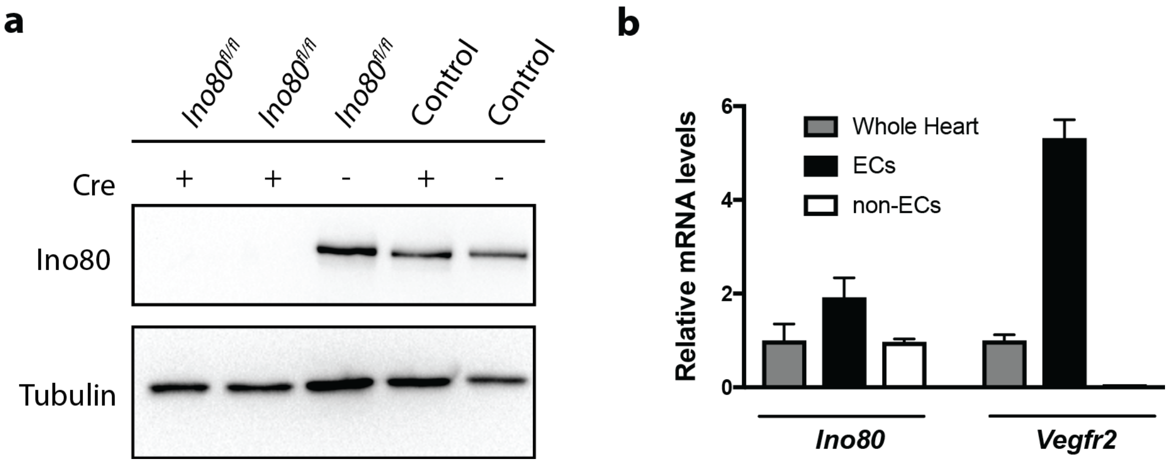

Supplementary Figure 1. *Ino80* deletion and expression. (**a**) Western blot analysis of lysates from primary mouse embryonic fibroblasts (MEFs) prepared from *Ino80 fl/fl* (n=3) and control embryos (n=2) infected with adenovirus (Cre -) or adenovirus expressing Cre recombinase (Cre +). Top panel is blot probed with anti-Ino80 antibody. Bottom panel is blot probed within anti-tubulin antibody. (**b**) qPCR for *Ino80* and *Vegfr2* and in endothelial and non-endothelial cells isolated from the heart (Sorted cells, n=3 hearts; whole hearts, n=3 hearts at e14.5). Error bars in graphs are standard deviation.

	Wild type		Het	CKO
Age (embryos)	*Tie2Cre- Ino80 fl/+* (25%)	*Tie2Cre- Ino80 fl/fl* (25%)	*Tie2Cre+ Ino80 fl/+* (25%)	*Tie2Cre+ Ino80 fl/fl* (25%)
e15.5 (91)	23%	25%	25%	26%
e16.5 (32)	34%	22%	31%	13%
e17.5 (18)	27%	22%	28%	22%
P1 (28)	28%	29%	35%	7%

Supplementary Figure 2. Embryonic lethality in *Tie2Cre;Ino80 fl/fl* embryos. Red indicates when the percentage of recovered conditional knockout (CKO) embryos was below the expected rates indicated in parentheses below genotypes.

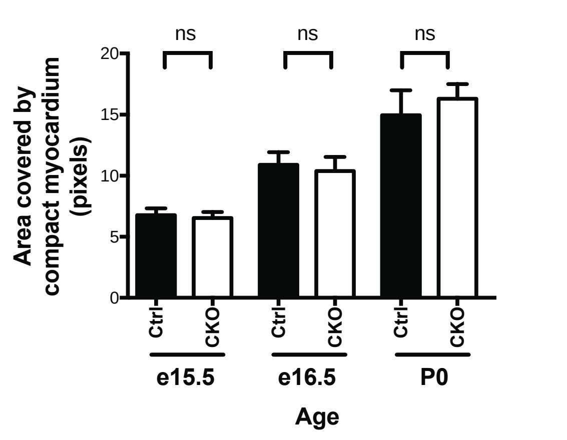

Supplementary Figure 3. Myocardial deletion of *Ino80* does not alter compaction. Deletion of *Ino80* in cardiomyocytes using the *Myh6Cre* deleter gene does not affect growth of the compact myocardium at indicated developmental stages. Error bars are standard deviation. (ns) Nonsignificant, evaluated by Student's *t*-test.

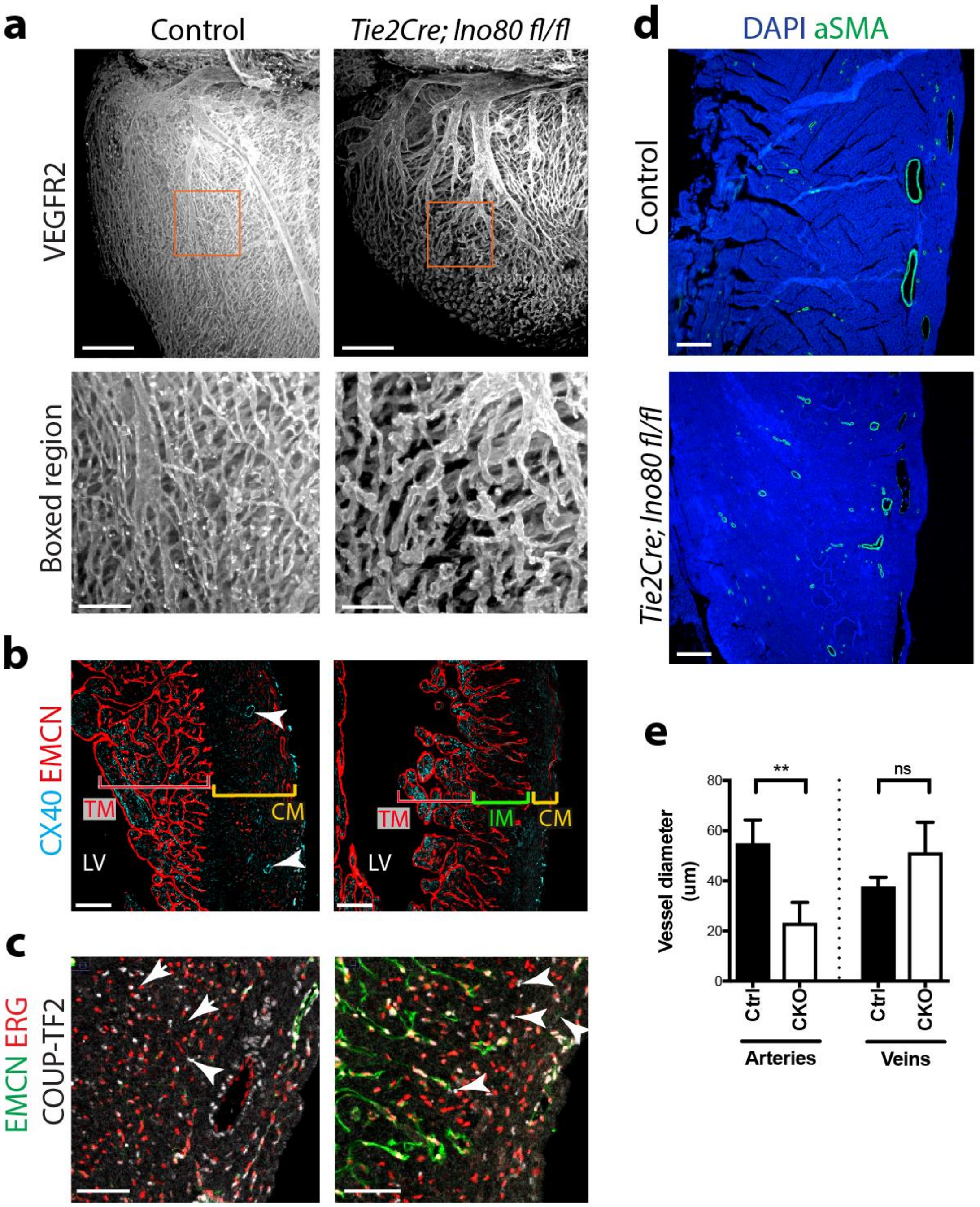

Supplementary Figure 4. Postnatal coronary development is defective in *Ino80* mutant hearts. (**a**) Whole mount confocal images of the coronary vasculature in P0. (control, n=3 hearts; mutant, n=3 hearts). Scale bars: 100 μm (low) and 25 μm (high magnification). (**b**) Tissue sections through P0 hearts revealed that CX40$^+$ arteries (arrowheads) are missing in mutants hearts and that the presence of the intermediate myocardium (IM) persists. (control, n=3 hearts; mutant, n=3 hearts). Scale bars: 100 μm. (**c**) Double labeling with pericyte (COUP-TF2) and endothelial (ERG) markers showed the presence of pericytes near coronary vessels (arrowheads) in control and mutant hearts. (control, n=3 hearts; mutant, n=3

hearts). Scale bars: 100 μm. (**d, e**) Immunofluorescence (**d**) and quantification (**e**) of arterial smooth muscle in P22 hearts indicate that surviving mutants have small arteries. Error bars in graphs are standard deviation. (control, n=4 hearts; mutant, n=5 hearts). (ns) Nonsignificant; (**) P < 0.01, evaluated by Student's *t*-test. Scale bars: 50 μm.

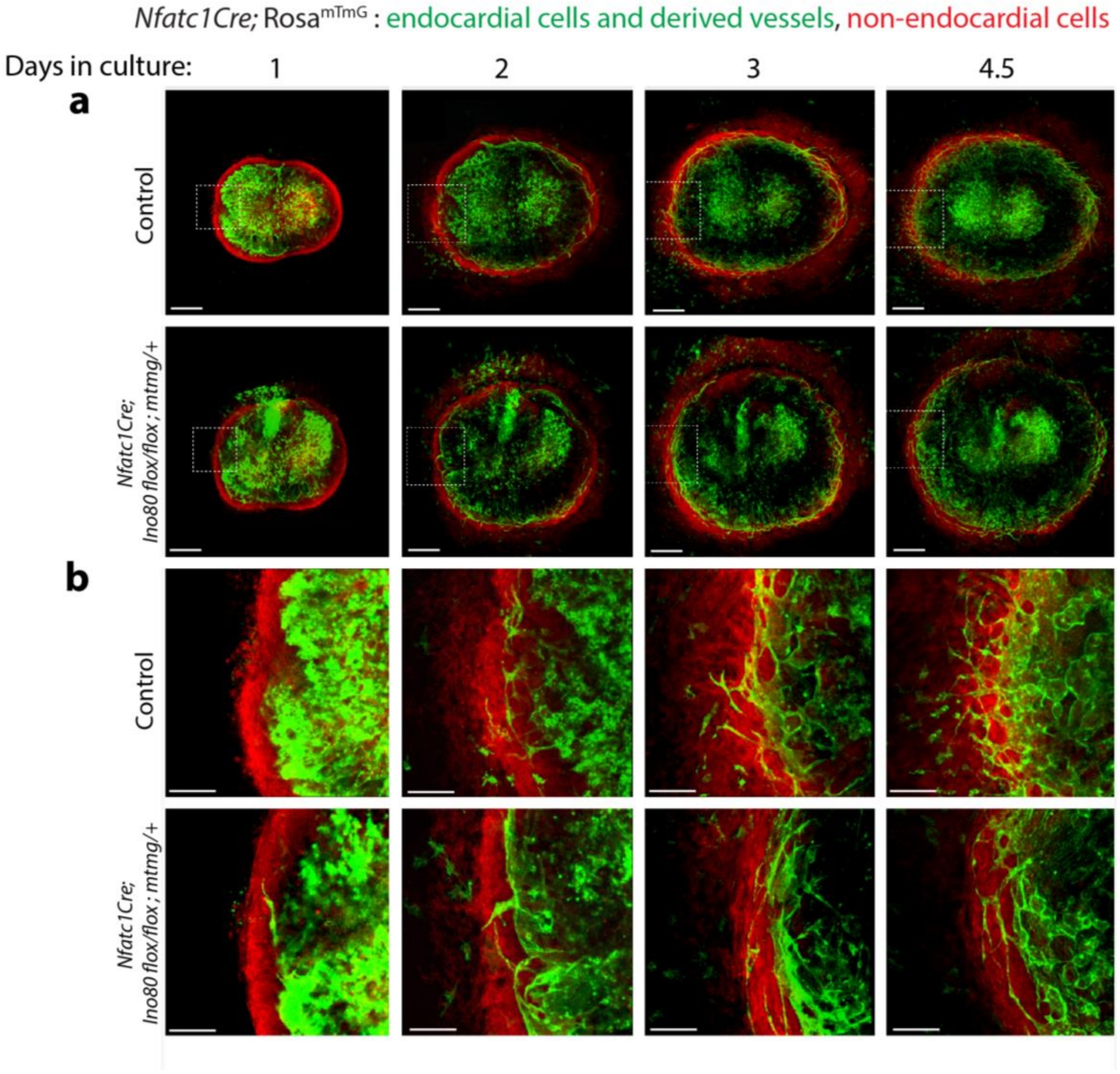

Supplementary Figure 5. Time lapse analysis of ventricle/endocardial cell cultures. Two representative ventricle cultures (one control and one *Nfatc1Cre;Ino80 flox/flox* mutant) imaged after the indicated number of days in cultures. Endocardial cells and their derivatives are labeled in green through *Nfatc1Cre*-mediated recombination of the Rosa^{mTmG} reporter allele. All other cells are labeled in red. Scale bars: 100 μm (a) and 25 μm (b).

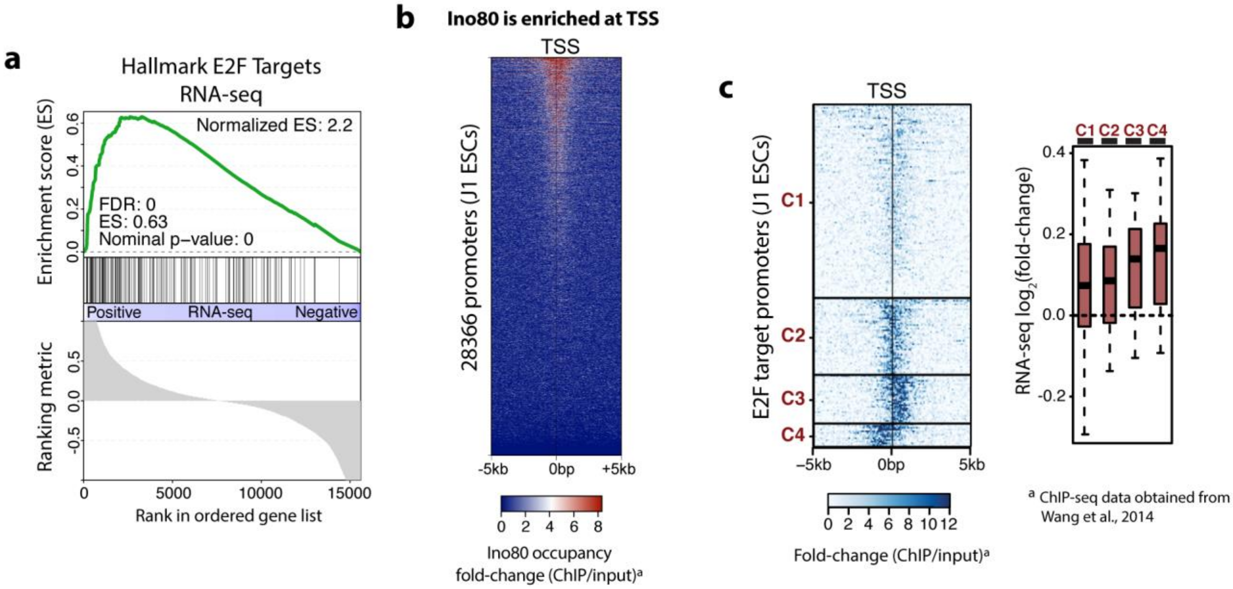

Supplementary Figure 6. Ino80 regulates E2F-target genes. (**a**) RNA-seq enrichment of hallmark E2F-target genes in *Tie2Cre;Ino80 fl/fl* hearts. False discovery rate (FDR) is adjusted p-value of enrichment. (**b**) Ino80 ChIP occupancy values (fold-change, IP/input) across 28,336 promoters within a 10kb window surrounding the transcription start site (TSS) of J1 embryonic stem cells (ESCs). (**c**) Left panel, Ino80 occupancy across individual genes are indicated by heatmap with k-means clustering. Right panel, boxplot shows RNA-seq expression trends for each of the 4 E2F-target clusters (C1-C4) in *Tie2Cre;Ino80 fl/fl* hearts. Wilcoxon rank sum p-values are <0.01 for each cluster compared to the genome-wide RNA-seq $\log_2$ fold change.

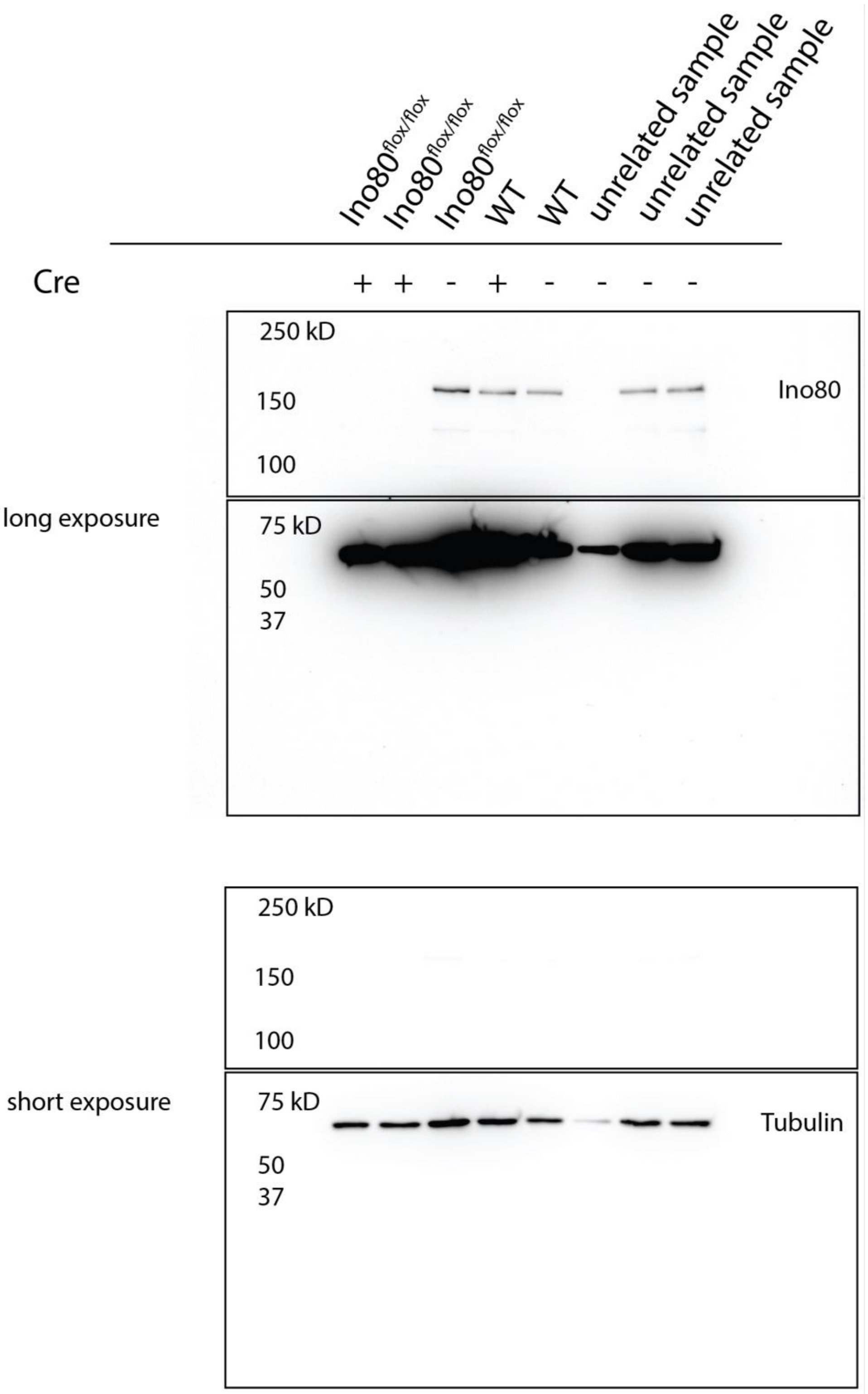

Supplementary Figure 7. Original immunoblot used for Supplementary Figure 1 indicated within the main manuscript.

Supplementary Table 1. Summaries of antibodies used for Immunostaining
Anybody information and working condition for immunostaining are provided in the table

Antibody	Manufacturer/Cat#	Dilutions/ Immunostaining condition			
		Whole mount	Paraffin section	Frozen section	Cell/ Explant
ENDOMUCIN	Santa Cruz (sc-65495)		1:250		
VE-cadherin	BD Biosciences	1:250		1:500	
CTNT	Developmental Studies Hybridoma Bank (CT3-c)	1:1000	1:1000	1:1000	
DACH1	Proteintech (10914-1-AP)	1:1000	1:1000	1:1000	
CX40	Alpha Diagnostic Intl. Inc., (CX40-A)	1:500	1:1000	1:1000	
hPROX1	R&D Systems (AF2727)		1:250		
COUP-TF2	Perseus Proteomics (PP-H7147-00)		1:1000		
Myomesin	Developmental Studies Hybridoma Bank, (mMaC myomesin B4)		1:1000	1:1000	
Myosin Heavy Chain	Alfa Aesar (BT 562)	1:1000			
NKX2.5	Santa Cruz (sc-8697)		1:250	1:250	1:250
ERG	Abcam (ab92513)	1:1000	1:1000	1:1000	1:1000
VEGFR2	R&D Systems (AF644)	1:250		1:250	
DAPI	Sigma Aldrich	1:1000	1:2000	1:2000	1:2000
