# Supplementary material for: Endothelial deletion of Ino80 disrupts coronary angiogenesis and causes congenital heart disease
Source: Nat Commun. 2018 Jan 25;9:368. doi: 10.1038/s41467-017-02796-3 (PMC5785521; doi:10.1038/s41467-017-02796-3)
Supplement: Supplementary file 2 — Description of Additional Supplementary Files [file 41467_2017_2796_MOESM2_ESM.pdf]

Description of Additional Supplementary Files

File Name: Supplementary Movie 1
Description: Time lapse imaging of control endothelial cells following experimental wounding. HUVEC monolayers treated with control siRNA were wounded with a scratch and imaged for 24 hours. Cells at the edge of the wound migrate to fill the available space. Movie is representative of 6 experimental repeats. Scale bar: 100 μm.

File Name: Supplementary Movie 2
Description: Time lapse imaging of *Ino80*-deficient endothelial cells following experimental wounding. HUVEC monolayers treated with *Ino80*-specific siRNA were wounded with a scratch and imaged for 24 hours. Migration of *Ino80*-depleted cells is delayed and less directional than controls while cell-cell contacts are also decreased. Movie is representative of 6 experimental repeats. Scale bar: 100 μm.
